# Cryo-EM of autoantibody-bound NMDA receptors reveals antigenic hotspots in an active immunization model of anti-NMDAR encephalitis

**DOI:** 10.1126/sciadv.aeb4249

**Published:** 2026-01-14

**Authors:** Junhoe Kim, Farzad Jalali-Yazdi, Brian E. Jones, Gary L. Westbrook, Eric Gouaux

**Affiliations:** ^1^ Vollum Institute, Oregon Health & Science University, 3232 SW Research Drive, Portland, OR 97239, USA.; ^2^ Howard Hughes Medical Institute, Oregon Health & Science University, 3232 SW Research Drive, Portland, OR 97239, USA.

## Abstract

Autoantibodies targeting synaptic membrane proteins are associated with autoimmune encephalitis manifested by seizures, psychosis, and memory dysfunction. Anti-*N*-methyl-d-aspartate receptor (NMDAR) encephalitis, a prototype of these autoimmune synaptic disorders, is unexpectedly common. Unfortunately, how the native repertoire of anti-NMDAR autoantibodies recognizes NMDARs and the precise locations of antigenic epitopes remain poorly understood. Here, we used an active immunization model that closely mimics the human disease to immunize adult mice with intact GluN1/GluN2A receptors, resulting in fulminant autoimmune encephalitis. Serum was collected at 6 weeks postimmunization for single-particle cryo–electron microscopy of GluN1/GluN2A receptors complexed with purified polyclonal anti-NMDAR autoantibody fragments. Native autoantibodies recognized two distinct binding sites on the GluN1 amino-terminal domain, which we confirmed using monoclonal antibodies bound to native NMDARs purified from mouse brain. Structural analysis of autoantibody-bound NMDAR complexes identified antigenic hotspots within the GluN1 amino-terminal domain. These hotspots provide potential targets for therapeutic intervention.

## Teaser

Anti-NMDAR autoimmune antibodies target small antigenic sites on GluN1 ATD.

## INTRODUCTION

Anti-*N*-methyl-d-aspartate (NMDA) receptors (NMDARs) mediate excitatory neurotransmission and are essential for the development and function of the nervous system (*1*). However, autoimmune antibodies can disrupt nervous system function by targeting neurotransmitter receptors at excitatory and inhibitory synapses, including NMDA (*2*), AMPA (*3*), γ-aminobutyric acid types A and B (GABA_A_ and GABA_B_) (*4*–*6*), and glycine (*7*) receptors. NMDARs in particular are common targets in autoimmune encephalitis (*8*). Anti-NMDAR autoantibodies are present not only in the serum and cerebrospinal fluid (CSF) of patients with anti-NMDAR encephalitis (*9*), but also in several other disorders including schizophrenia (*10*) and systemic lupus erythematosus (SLE) (*11*). The trigger that initiates the autoimmunity is not clear, but may involve viral infections or ovarian tumors, leading to the production of anti-NMDAR autoantibodies (*8*, *12*). Such patients present with a neuropsychiatric syndrome including hallucinations, seizures, psychosis, memory deficits, and even catatonia (*2*, *13*).

In mice, passive transfer of patient-derived immunoglobulin G (IgG) induces memory deficits and behavioral abnormalities (*14*, *15*). However, active immunization of mice with intact NMDARs incorporated in proteoliposomes more closely mimics the neurological changes, including seizures, as seen in human patients (*16*). Serum or CSF from human cases or mouse models of NMDAR encephalitis reduces NMDAR surface expression and postsynaptic currents (*16*, *17*), which suggests a pathogenic role for antibody-induced receptor cross-linking, internalization, and degradation (*8*). Thus, an understanding of the molecular mechanisms underlying the role of anti-NMDAR autoantibodies is essential for successful therapies.

NMDARs assemble as heterotetramers, comprising two obligatory glycine-binding GluN1 subunits and two glutamate-binding GluN2A-D and/or glycine-binding GluN3A-B subunits (*1*). All subunits share a modular architecture and are organized into layers: the amino-terminal domain (ATD), ligand-binding domain (LBD), and transmembrane domain (TMD) (*18*). The ATD plays a critical role in modulating channel activity through its interactions with the LBD via binding of allosteric modulators and cations such as Zn^2+^ and protons (*1*, *19*). Recent studies demonstrated that anti-NMDAR autoantibodies primarily target the ATD of the GluN1 subunit (*20*, *21*). Likewise, structural studies have reported that patient-derived monoclonal antibodies (mAbs) bind to the GluN1 ATD (*22*, *23*). However, these structures capture several examples of autoantibody-receptor complexes and likely do not represent the potential diversity of autoantibody-receptor interactions. Furthermore, the specific regions within GluN1 that confer high antigenic potential remain incompletely defined.

Here, we elucidated the cryo–electron microscopy (cryo-EM) structures of GluN1/GluN2A receptors in complex with polyclonal antibody (pAb) fragments that represent the autoantibody repertoire from anti-NMDAR encephalitis mice. Using mAbs that target GluN1 and GluN2A subunits, we further resolved the molecular interactions at the autoantibody-receptor interfaces in native NMDARs purified from mouse brain. By comparing multiple NMDAR-autoantibody complex structures, we defined discrete regions (hotspots) on the GluN1 ATD targeted by autoantibodies. Together, our findings define the key antigenic epitopes within the GluN1 ATD that underlie autoantibody recognition.

## RESULTS

### Generation of NMDAR-targeting autoantibodies from a mouse model

**Fig. 1.**
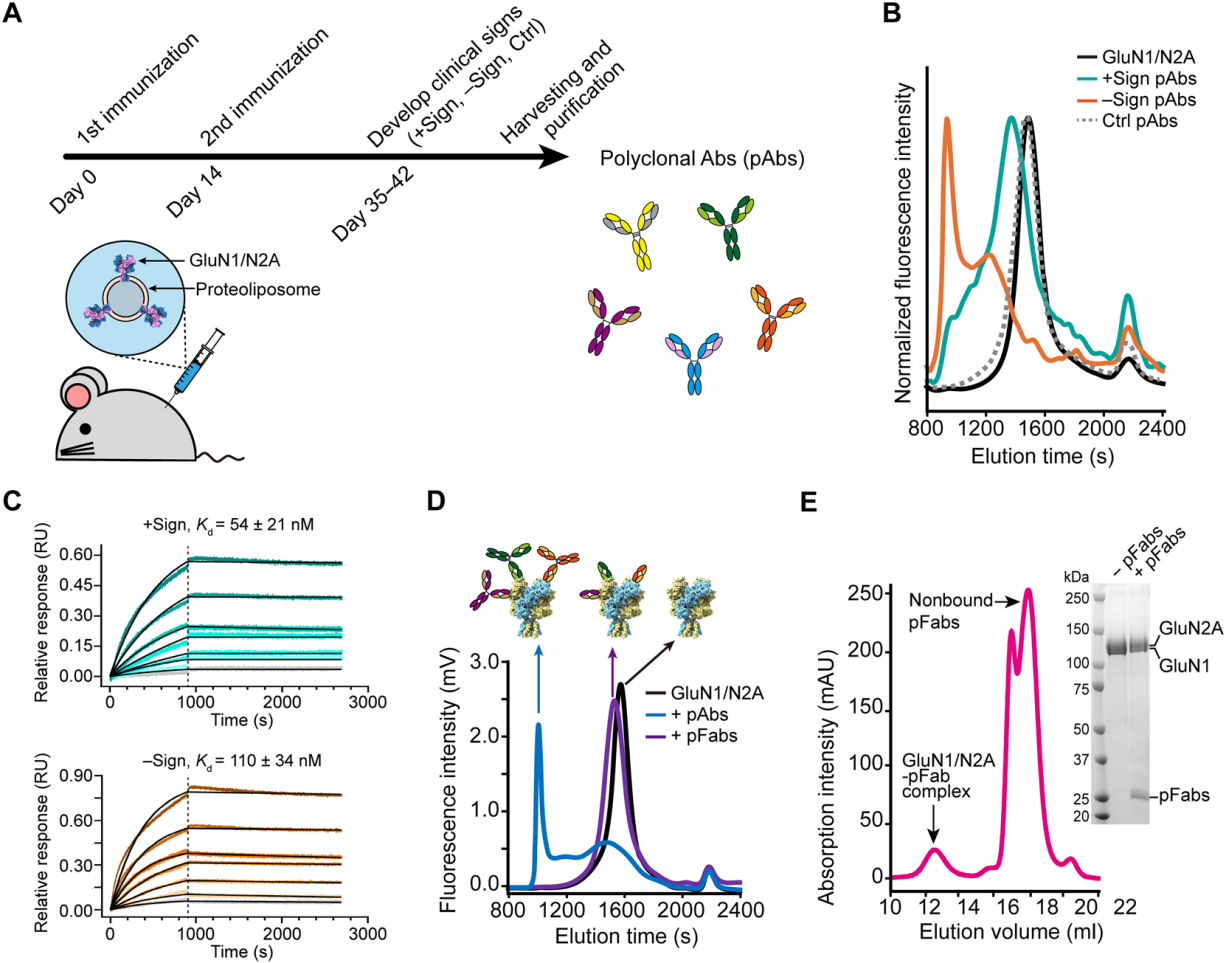
Generation of NMDAR autoimmune antibodies. (**A**) Schematic workflow illustrating the immunization protocol used to generate anti-NMDAR autoantibodies in mice. (**B**) FSEC profiles of GFP-tagged GluN1/GluN2A receptors alone and in complex with the indicated pAbs isolated from +Sign or −Sign mice. (**C**) Measurement of pAb binding to GluN1/GluN2A receptors from the +Sign or −Sign mice (see Materials and Methods). Data are means ± SEM. (**D**) FSEC profiles of GluN1/GluN2A receptors in complex with pAbs and their corresponding pFabs, alongside schematic illustrations of the complexes. mV, millivolt. (**E**) SEC profile of the GluN1/GluN2A-pFab complex assembled under a subsaturating condition of pFabs (see also fig. S1, E and F). Inset: SDS-PAGE gel image showing the purified sample. Ctrl, control; *K*_d_, dissociation constant; RU, response units; mAU, milli-absorbance units.

Active immunization of mice with liposomes harboring fully assembled GluN1/GluN2 NMDARs, deemed proteoliposomes, induces characteristic encephalitic signs as also observed in human patients, thereby establishing a robust mouse model for studying anti-NMDAR encephalitis (*16*). Using this model, we sought to elucidate how the autoantibody repertoire recognizes NMDARs using single-particle cryo-EM. We immunized mice (*n* = 12) with GluN1/GluN2A-containing proteoliposomes on day 0 and day 14 (Fig. 1A and fig. S1, A to C) and assessed their behavioral changes as determined in the previous study (see Materials and Methods) (*16*). Upon analysis, the mice were divided into two groups based on the presence or absence of encephalitic signs, such as hyperactivity, circling, and seizures, and denoted by “+Sign” (*n* = 7) and “−Sign” (*n* = 5), respectively. We purified a pool of pAbs from these mice after 6 weeks of postimmunization. We also purified the native repertoire of IgG from adult mice (*n* = 4) injected with liposomes without NMDARs (liposome-only), which had no encephalitic signs, thus representing a control group. As part of our first assay to measure the presence of antibodies that bind to the NMDAR, we used GluN1/GluN2A receptors fused to green fluorescent protein (GFP) and measured their elution from a size exclusion chromatography (SEC) column, following receptor fluorescence (FSEC) (*24*). Supplementing the receptor complex with the purified pAbs from the +Sign and −Sign mice shifted the elution of the receptor complex to an earlier volume, indicating the formation of receptor-autoantibody complexes (Fig. 1B). By contrast, the pAbs isolated from the liposome-only control mice did not alter the elution volume, showing no detectable antibodies reacting to GluN1/GluN2A receptors. Although the mice in the −Sign group did not develop recognizable encephalitic signs, the pAbs from this group shifted the receptor peak even more than those from +Sign (Fig. 1B). This larger shift of the receptor complex could be due to binding of multiple antibodies, perhaps indicating that binding sites of pAbs are more diversified in −Sign mice. In contrast, the smaller shift observed with pAbs from the +Sign mice suggested that fewer pAbs bind to the receptor simultaneously. Some antibodies from pAbs from +Sign and −Sign bound to GluN1/GluN2A receptors at high affinity [dissociation constant (binding affinity), *K*_d_] of 54 ± 21 and 110 ± 34 nM, respectively (Fig. 1C). These observations are consistent with previous findings that proteoliposome-immunized mice lacking clinical signs have histological features and anti-NMDAR autoantibodies (*16*).

To obtain complexes of the GluN1/GluN2A receptor and pAbs without formation of oligomerized clusters, we digested the antibodies from +Sign mice with papain to yield Fab fragments (pFabs), which were subsequently isolated from Fc fragments via protein A/G chromatography (fig. S1D; see Materials and Methods). The resulting pFab proteins showed dramatically reduced aggregation in complexes with GluN1/GluN2A receptors in FSEC (Fig. 1D), as judged by the absence of early eluting species. Some binding sites could interact with several antibodies, with high-affinity antibodies dominating. Therefore, we investigated an antibody concentration below saturation to allow binding of both low- and high-affinity antibodies. We assessed the extent of peak shifts across various receptor-to-pFab ratios, using a 14- to 420-fold excess of pFabs relative to the receptor concentration. From this analysis, we empirically determined that a 1-to-140 ratio yields a sufficient amount of subsaturated receptor complexes for downstream experiments, given the limited antibody quantities isolated from mice (fig. S1, E and F). We then purified GluN1/GluN2A-pFab complexes by SEC, from a large amount of unbound pFabs (Fig. 1E), which are presumably weak binding or non–NMDAR-targeting antibodies derived from the mouse immune system.

### Structural determination of NMDAR-pFab complexes

**Fig. 2.**
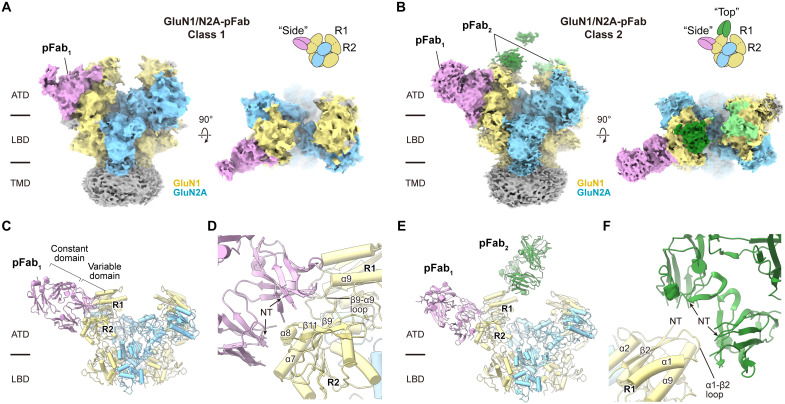
Structures of NMDAR-pFab complexes. (**A** and **B**) Cryo-EM density maps of GluN1/GluN2A-pFab class 1 (A) and class 2 (B), with schematics illustrating the “side”’ and “top” antibody binding modes. Color scheme: GluN1 (yellow), GluN2A (blue), pFab_1_ (pink), pFab_2_ (green), and detergent micelle (gray). (**C**) Structure of the ATD-LBD layer in class 1, showing the GluN1/GluN2A receptor in complex with one side-bound pFab_1_. (**D**) Enlarged view of the pFab_1_ binding site demonstrating the structural elements of GluN1 involved in the interaction. NT, N terminus. (**E**) Structure of the ATD-LBD layer in class 2, showing the GluN1/GluN2A receptor in complex with one side-bound pFab_1_ and one top-bound pFab_2_. (**F**) Enlarged view of the pFab_2_ binding site showing the GluN1 structural elements involved in the interaction.

Next, we carried out single-particle cryo-EM analysis of the GluN1/GluN2A-pFab complexes in the presence of the saturating ligands glycine and glutamate (fig. S2). Two-dimensional (2D) class average images revealed pFab densities at the ATD. Further image processing with extensive 3D classifications identified multiple subclasses of GluN1/GluN2A-pFab complexes, which were most well resolved into two classes: class 1 with pFabs bound laterally to the receptor (pFab_1_; “side”), parallel to the membrane plane, and class 2 with multiple pFabs including the ones bound to the receptor along the vertical axis (pFab_2_; “top”). Class 1 and class 2 were then independently refined without symmetry application and yielded nominal 4.07- and 3.92-Å resolution reconstructions, respectively (Fig. 2, A and B, and Table 1), which enabled definition of the extracellular ATDs and LBDs, yet were insufficient to fit small side chains. Although pFab densities were discernible, they were less well resolved than the receptor, limiting our ability to define the structures of the complementarity-determining regions (CDRs). We speculate that the lower resolution of the CDRs is likely due to heterogeneous interactions of pAbs at their respective binding interfaces. Notably, we did not find antibodies against the LBD or the TMD, likely because these regions of the receptor offer far less solvent-accessible surface area. Because there were no other classes observed in a distinct antibody-bound conformation, we propose that these two types of pFabs mediate the most of the NMDAR-autoimmune antibody interactions.

**Table 1.** Statistics of cryo-EM data collection and model refinement. FSC, Fourier shell correlation.

	GluN1/N2A- pFabs class 1	GluN1/N2A- pFabs class 2	Native 3 Fab-bound GluN1/N2A/N2B class 1	Native 3 Fab-bound GluN1/N2A/Nx class 2	Native 4 Fab-bound GluN1/N2A class 3		Native 2 Fab-bound GluN1/GluN2B class 4	Native 2 Fab-bound GluN1/GluN2x class 5	Native GluN1/N2A-5F11/3D2 local ATD dimer	GluN1/N2A-3D2	GluN1/N2A-3D2 local ATD dimer
	EMD-72076 PDB 9PZQ	EMD-72077 PDB 9PZR	EMD-72078 PDB 9PZS	EMD-72079	EMD-72080 PDB 9PZT		EMD-72081 PDB 9PZU	EMD-72082	EMD-72083 PDB 9PZV	EMD-72084 PDB 9PZW	EMD-72085 PDB 9PZX
**Data collection and processing**											
Microscope	FEI Titan Krios	FEI Titan Krios	FEI Titan Krios	FEI Titan Krios	FEI Titan Krios		FEI Titan Krios	FEI Titan Krios	FEI Titan Krios	FEI Titan Krios	FEI Titan Krios
Camera, energy filter	K3, BioQuantum	K3, BioQuantum	K3, BioQuantum	K3, BioQuantum	K3, BioQuantum		K3, BioQuantum	K3, BioQuantum	K3, BioQuantum	K3, BioQuantum	K3, BioQuantum
Voltage (kV)	300	300	300	300	300		300	300	300	300	300
Magnification	29,000	29,000	64,000	64,000	64,000		64,000	64,000	64,000	105,000	105,000
Energy filter slit width (eV)	20	20	20	20	20		20	20	20	20	20
Dose rate (e^−^ pix^−1^ s^−1^)	15	15	8	8	8		8	8	8	23	23
Total electron exposure (e^−^/Å^2^)	45	45	65	65	65		65	65	65	50	50
Number of movie frames	40	40	120	120	120		120	120	120	45	45
Pixel size (Å/pix)	0.798 (0.399)	0.798 (0.399)	1.394 (0.697)	1.394 (0.697)	1.394 (0.697)		1.394 (0.697)	1.394 (0.697)	1.394 (0.697)	0.830 (0.415)	0.830 (0.415)
Defocus range (μm)	−1.4 to −2.5	−1.4 to −2.5	−1.0 to −3.0	−1.0 to −3.0	−1.0 to −3.0		−1.0 to −3.0	−1.0 to −3.0	−1.0 to −3.0	−1.0 to −2.5	−1.0 to −2.5
Number of micrographs	4,119	4,119	9,851	9,851	9,851		9,851	9,851	9,851	6,721	6,721
Number of processed particles	49,231	54,790	296,585	32,759	26,341		163,121	109,325	624,529	183,438	366,876
Symmetry imposed	C1	C1	C1	C1	C1	C2	C1	C1	C1	C1	C1
B-factor (Å^2^)	60.3	60.6	106.0	371.3	241.1	204.7	332.9	226.8	136.1	107.8	59.9
Map resolution (Å), FSC = 0.143	4.07	3.92	4.20	6.56	6.14	5.98	5.36	5.05	3.92	3.43	2.76
**Model refinement and validation**											
Model resolution (Å), FSC = 0.5	8.1	8.5	8.1		8.9		8.6		7.4	3.6	3.9
Model composition											
Atoms	21,985	23,601	24,319		24,703		22,362		8,069	27,539	6,120
Protein residues	3,010	3,362	3,415		3,446		3,152		1,145	3,497	783
Ligands	0	0	16		25		12		11	17	6
Bond (RMSD)											
Length (Å)	0.004	0.005	0.003		0.003		0.004		0.002	0.004	0.003
Angle (°)	0.750	0.806	0.580		0.608		0.622		0.518	0.576	0.576
Validation											
MolProbity score	1.78	1.84	1.90		1.94		1.95		1.95	1.76	1.93
Clashscore	8.01	9.42	11.90		13.22		12.93		7.62	6.45	5.15
Ramachandran plot											
Outliers (%)	0.07	0.12	0.00		0.00		0.00		0.00	0.00	0.00
Allowed (%)	4.94	4.82	4.42		4.46		4.65		5.38	6.07	6.11
Favored (%)	94.99	95.06	95.58		95.54		95.35		94.62	93.93	93.89
Rotamer outliers (%)	0.09	0.10	0.14		0.18		0.15		1.63	0.44	2.14
Cβ outliers (%)	0.00	0.00	0.00		0.00		0.00		0.00	0.00	0.00
CC (model versus map, mask)	0.61	0.56	0.54		0.68		0.63		0.53	0.79	0.71

The ATDs of NMDAR subunits form a bilobe structure with the R1 and R2 lobes connected with linkers and play crucial roles in modulation of channel function in response to divalent ions, pH changes, and allosteric modulators (*1*, *18*, *19*, *25*). In the GluN1/GluN2A-pFab structures, both pFabs bind to the GluN1 subunit via the R2 lobe for pFab_1_ and the R1 lobe for pFab_2_ (Fig. 2, C to F). pFab_1_ displays close contacts with α7, α8, the β9-α9 loop, and the β10-β11 loop in the GluN1 R2 lobe, suggesting potential interactions with these structural elements. For pFab_2_, the cryo-EM densities of their constant domains remain unresolved, and one pFab_2_ density is more prominent than the other, which implies a possible clash or unfavored geometry between these antibodies. We thus further processed the map with focusing on one side of the ATD heterodimer and better resolved the pFab_2_ density that allowed structural modeling (fig. S2). pFab_2_ makes close contact with the vertical edge of the GluN1 R1 lobe via structural elements of the amino-terminal region and the α1-β2 loop. Together, these polyclonal Fab fragments derived from the anti-NMDAR encephalitis mice recognized the most exposed regions of the extracellular part of the receptors, especially in the GluN1 ATD. Therefore, we suggest that the GluN1 ATD provides accessible sites for autoimmune antibody recognition, which is aligned with previous observations that autoimmune antibodies derived from patients with anti-NMDAR encephalitis primarily target the GluN1 subunit (*20*, *21*).

### Characterization of mAbs targeting GluN1 and GluN2A subunits

**Fig. 3.**
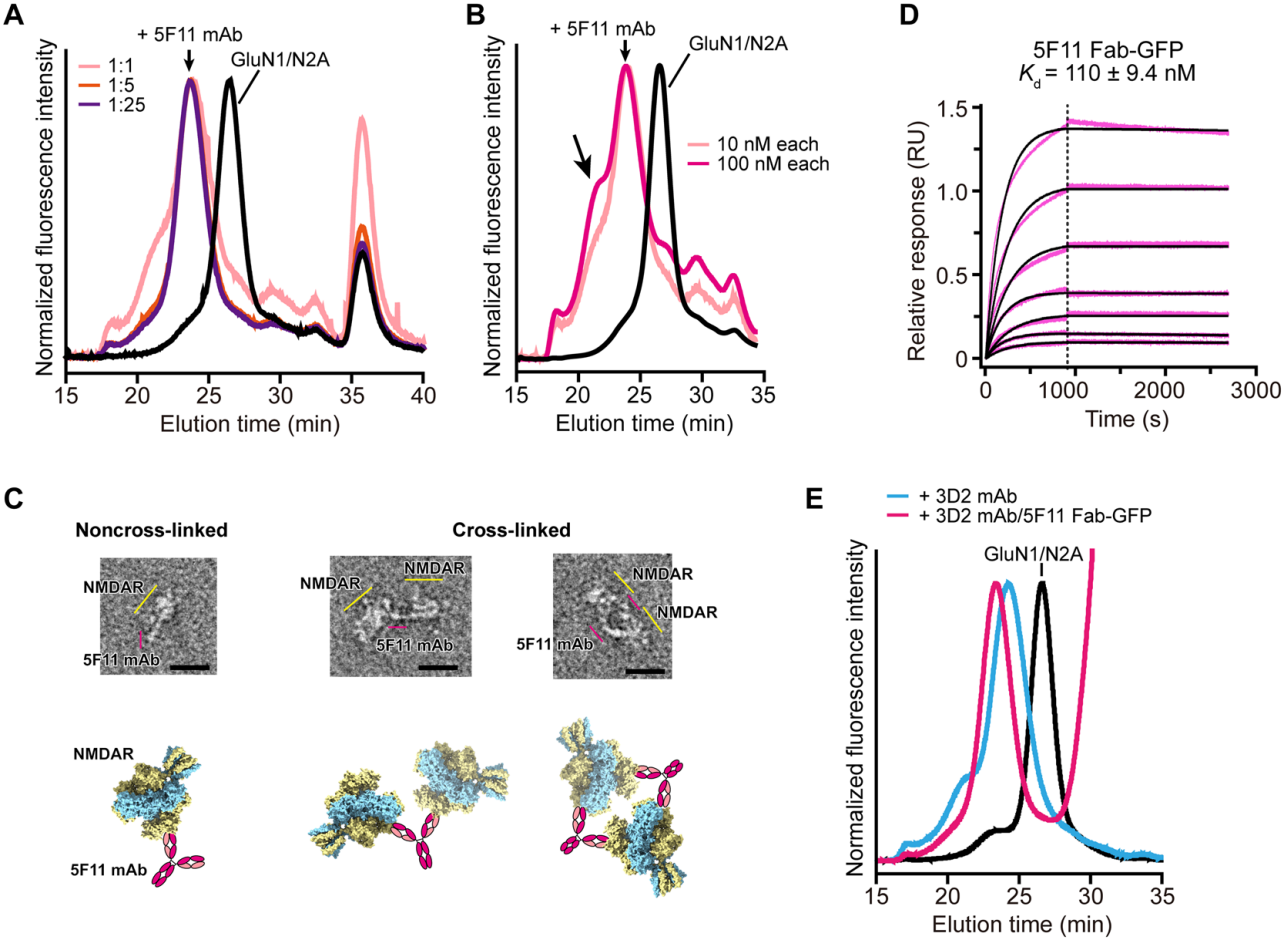
Characterization of mAbs targeting GluN1 and GluN2A subunits. (**A**) FSEC profiles of GluN1/GluN2A receptors alone (10 nM) and in complex with the indicated ratio of 5F11 mAb. (**B**) FSEC profiles of GluN1/GluN2A receptors bound to 5F11 at a 1:1 ratio at the indicated concentrations (nM), showing a subset of receptors cross-linked and detected at an earlier elution volume (arrow). (**C**) Negative-stain EM images of GluN1/GluN2A receptors in the presence of 5F11 mAb. Schematics illustrate the models of GluN1/GluN2A-5F11 mAb complexes as discerned from the micrographs. Scale bars, 25 nm. (**D**) Binding affinity measurements of GluN1/GluN2A receptors with 5F11 Fab-GFP (see Materials and Methods). Data are means ± SEM. (**E**) FSEC profiles of GluN1/GluN2A in complex with the indicated antibodies, demonstrating simultaneous binding of 5F11 and 3D2 to the receptors.

We also used active immunization with intact NMDARs in proteoliposomes to develop a high-affinity GluN1-specific mAb, deemed 5F11 (*26*), which binds to a 3D epitope of GluN1/GluN2A receptors (Fig. 3A). Supplementation of 5F11 mAb to receptors in 1:1 molar ratio produces a leading peak at a higher molecular-weight position, suggestive of a supercomplex formation by linking multiple NMDARs with 5F11 mAbs (Fig. 3B). In negative stain EM, we discerned that 5F11 mAbs bind to GluN1/GluN2A ATDs (Fig. 3C), in a fashion analogous to pFab_1_ in the GluN1/GluN2A-pFab complex. In some cases, two receptors were captured by one 5F11 mAb via its bivalent binding sites. Because NMDAR clustering (*8*) is a known feature of anti-NMDAR encephalitis, we speculate that antibody-mediated receptor cross-linking represents a possible underlying mechanism.

To enable nonclustered, homogeneous complex formation of 5F11 and NMDARs, we designed the recombinant expression of a 5F11 Fab fragment fused to GFP (5F11 Fab-GFP) (*26*). 5F11 Fab-GFP displayed high affinity to GluN1/GluN2A receptors with *K*_d_ of 110 nM (Fig. 3D). We also developed a GluN2A-specific high-affinity mAb, 3D2 (*26*), that recognizes a 3D epitope of the receptor. Supplementation of 5F11 Fab-GFP “super-shifts” the elution volume of the GluN1/GluN2A-3D2 mAb complex (Fig. 3E), showing simultaneous binding of these antibodies to the NMDAR complex.

### Structures of native NMDAR-autoantibody complexes reveal antigenic surfaces

**Fig. 4.**
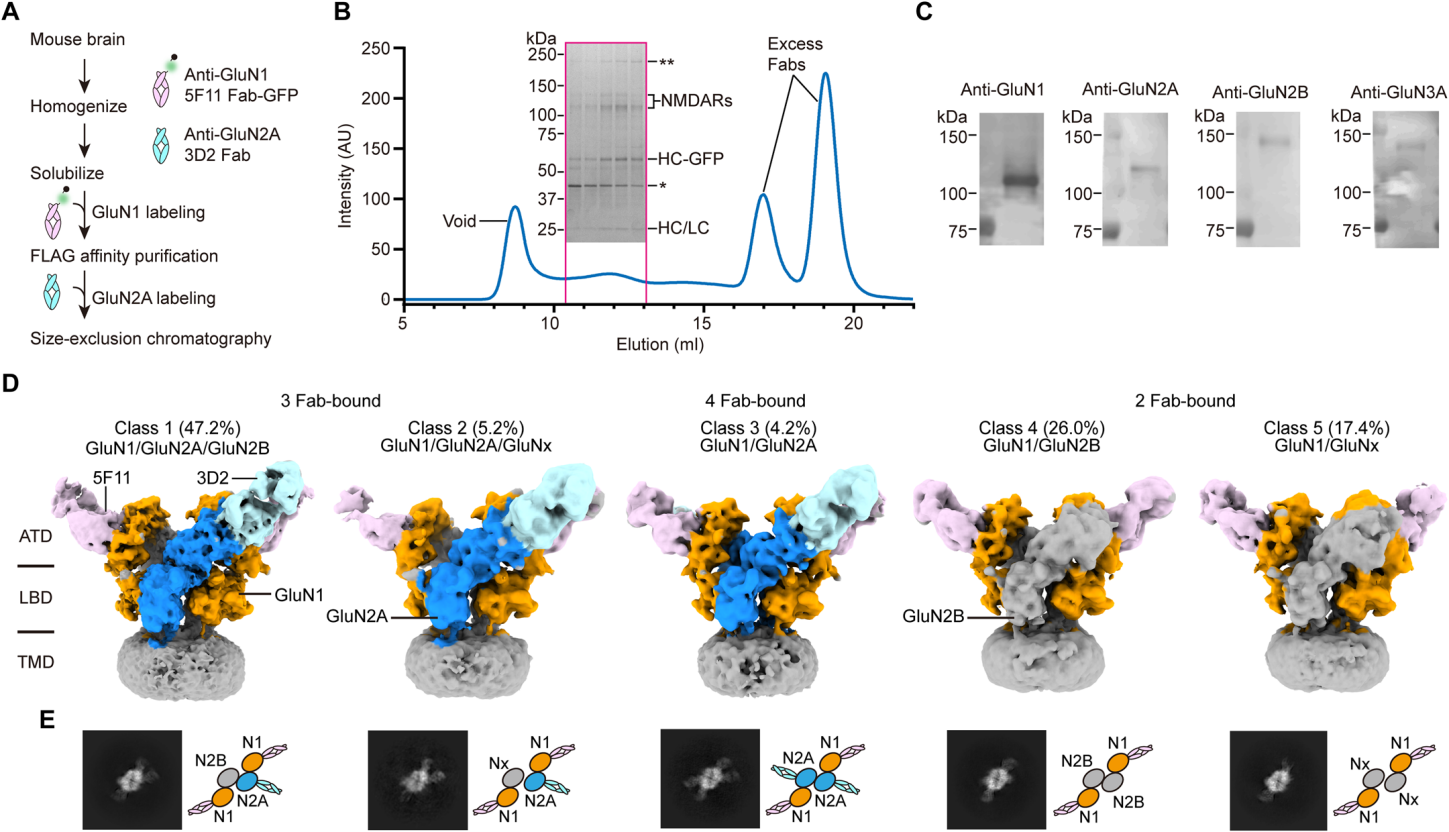
Cryo-EM structures of native NMDAR-autoantibody complexes reveal antigenic surfaces. (**A**) Schematic of the workflow for purifying native NMDARs from mouse brain using subunit-specific antibodies. (**B**) SEC profile showing native NMDARs in complex with 5F11 Fab-GFP and 3D2 Fab. Inset: SDS-PAGE gel image of the purified receptor-antibody complexes. Unidentified protein bands are marked with asterisks. HC, heavy chain; LC, light chain. (**C**) Western blot analysis of the purified native receptor complexes, detecting GluN1, GluN2A, GluN2B, and GluN3A subunits. (**D**) Cryo-EM density maps of native NMDARs in different antibody-bound states. Color scheme: GluN1 (orange), GluN2A (blue), 5F11 Fab (pink), 3D2 Fab (light blue), GluN2B (or GluNx) and micelle (gray). (**E**) Representative 2D class averages and schematics from top-down views of each class, showing conformational differences in the ATD layer.

We next sought to understand how 5F11 and 3D2 mAbs that target GluN1 and GluN2A subunits bind to native NMDARs and recognize their antigenic epitopes. We used the whole brain from mice aged 29 to 42 days and purified NMDARs by immunoaffinity purification with 5F11 Fab-GFP (Fig. 4A). The NMDAR-antibody complexes exhibited a broadened SEC profile and distributed bands in SDS–polyacrylamide gel electrophoresis (SDS-PAGE), indicating that 5F11 Fab-GFP successfully captured multiple NMDAR subtypes and their alternatively spliced forms (Fig. 4B). Western blot and FSEC analyses showed that ~80 to 90% of native NMDARs were solubilized and effectively captured during the purification process (fig. S3, A to C). The purified native NMDARs were largely composed of GluN1, GluN2A, and GluN2B subunits based on Western blot analysis (Fig. 4C). GluN3A subunits were also detected at intermediate levels, supporting recent findings of their sustained expression in the juvenile mouse brain (*27*, *28*). No detectable signal was observed for GluN2C and GluN2D subunits in the Western blot analysis (fig. S3, D and E), likely due to relatively low affinity of the available antibodies, combined with the low proportion of these subunits within the total native NMDAR population.

NMDARs reside at excitatory glutamatergic synapses, where high concentrations of glutamate are transiently released into the synaptic cleft and the co-agonist glycine or d-serine is present for receptor activation (*1*, *29*). Thus, we added glycine and glutamate under a saturating condition (1 mM each) to the purified sample and collected a cryo-EM dataset (see Materials and Methods). Two-dimensional class average images show the EM densities of both 5F11 and 3D2 Fabs at the ATD layer of the receptor (fig. S4). To separate the NMDAR subtypes based on the antibody-bound states, we carried out extensive heterogeneous refinement and 3D classifications, focusing on the ATD region. After refinement of individual subclasses, we resolved three distinct antibody-bound states of native NMDARs in five different classes—three Fab-bound (triheteromeric GluN1/GluN2A/GluNx; class 1 and class 2), four Fab-bound (diheteromeric GluN1/GluN2A; class 3), and two Fab-bound (GluN1/GluNx; class 4 and class 5)—in a resolution range of 4.20 to 6.56 Å (Fig. 4, D and E, and Table 1). Class 2 and class 5 show slightly extended ATD conformations, suggesting conformational variants or different receptor subtypes, although interpretation is limited by the low-resolution reconstruction. By contrast, the ATDs in class 1, class 3, and class 4 are tightly packed, in a conformation that is consistent with those of previously studied GluN1/GluN2A and GluN1/GluN2B receptors (*19*, *30*). Given that GluN2A and GluN2B are the most abundant subunits in the purified sample as shown in the Western blot, class 1 and class 4 likely represent triheteromeric GluN1/GluN2A/GluN2B and diheteromeric GluN1/GluN2B, respectively (fig. S5). Notably, according to the particles used in the final reconstruction of each class, ~47% of native NMDARs are GluN1/GluN2A/GluN2B receptors (class 1), consistent with previous observations that triheteromeric NMDARs are the most abundant form in the brain (*31*–*34*).

All native NMDAR classes display tetrameric arrangement with two GluN1 subunits occupying the A/C positions and two GluN2 and/or GluN3 subunits occupying the B/D positions (*35*, *36*), distinguished by the cryo-EM densities of bound antibodies. The 5F11 and 3D2 Fabs bind to the R2 lobe of GluN1 ATD and the R1 lobe of GluN2A ATD, respectively, without structural hindrance. Notably, the 5F11 Fab binding site on the GluN1 subunit is distant from the region where 21 amino acids are alternatively spliced in exon 5 (fig. S6A) (*37*, *38*). Therefore, the purified sample is unbiased and may represent the full range of native NMDAR diversity in the mouse brain. The bilobed clamshell-like LBD structures, consisting of D1 and D2 lobes (*39*), display the closed conformations in all classes (fig. S6B), consistent with the previously reported glycine- or glutamate-bound structures (*19*, *30*, *40*–*46*). The EM densities of TMDs, however, were comparatively less resolved, limiting determination of the channel conformation(s) in the current agonist- and Fab-bound condition.

### Molecular determinants of autoantibody-epitope interactions

**Fig. 5.**
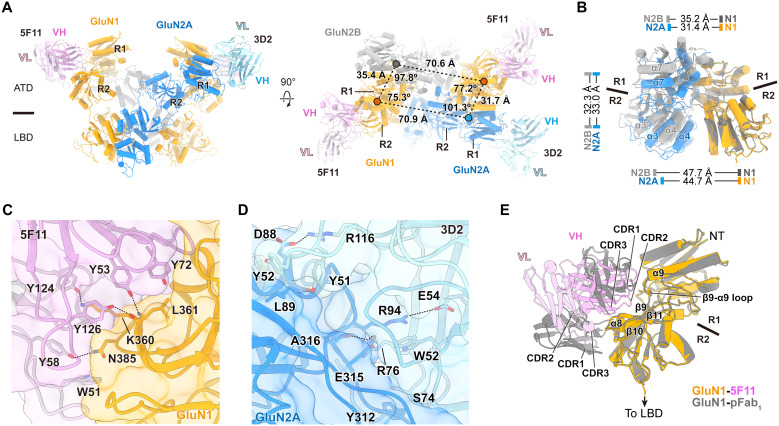
Autoantibody-epitope interactions. (**A**) Structure of the ATD-LBD layer of the native GluN1/GluN2A/GluN2B receptor in complex with 5F11 and 3D2 Fabs. Distances and angles were measured using the COM of the ATD in each subunit, revealing asymmetric arrangement of the ATD in the triheteromeric receptor. Color scheme: GluN1 (orange), GluN2A (blue), GluN2B (gray), 5F11 (pink), and 3D2 (light blue). (**B**) Superposition of the “local” GluN1-GluN2A and GluN1-GluN2B ATD dimer structures, aligned at GluN1 ATDs, demonstrating differences in COM distances between their respective R1 and R2 lobes. (**C**) Structure of the GluN1-5F11 interface overlaid with a surface representation (transparent) showing residues involved in the interactions. Dotted lines indicate hydrogen bonds. (**D**) Structure of the GluN2A-3D2 interface overlaid with a surface representation (transparent). (**E**) Superposition of the pFab_1_- and 5F11-bound GluN1 ATD structures, aligned at GluN1, showing substantial overlap in the GluN1 regions that involve antibody recognition.

We next focused on the conformation of antibody-bound ATDs in the best-resolved triheteromeric GluN1/GluN2A/GluN2B receptor, which represents the most abundant NMDAR subtype in the mouse brain used in this study (P28-42). The ATDs of the triheteromeric receptor exhibit an asymmetric arrangement, as revealed by distances and angles measured using the center of mass (COM) of ATD in each subunit (Fig. 5A). Superposition of the “local” ATD dimers, aligned on the GluN1 subunits, reveals a more extended conformation of the ATD heterodimer in the GluN1-GluN2B assembly compared to that in the GluN1-GluN2A assembly (Fig. 5B).

To better resolve the molecular interactions between the receptor and antibodies, we combined all particles from the five classes and performed focused refinement on one side of the ATD heterodimer, including the variable domains of the 5F11 and 3D2 Fabs (fig. S4). The resulting “local” GluN1/GluN2A ATD-5F11/3D2 complex was resolved at 3.92 Å resolution (fig. S5G and Table 1), allowing structural modeling of key residues involved in GluN1-5F11 interactions (fig. S7, A and B). In addition, we collected an independent cryo-EM dataset of the recombinant GluN1/GluN2A-3D2 complex in the same agonist-bound condition and, with focused refinement on the ATD-antibody region, resolved the density map at 2.76 Å resolution, which allowed us to build most of the side chains at the GluN2A-3D2 interface (figs. S7, C and D, and S8; and Table 1). The conformation of this high-resolution “local” GluN1/GluN2A-3D2 ATD dimer closely resembles [Cα root mean square deviation (RMSD) = 0.891 Å] the corresponding region in the native GluN1/GluN2A/GluN2B-5F11/3D2 complex (fig. S9), providing structural guidance for analyzing the GluN2A-3D2 interface within the context of 5F11-bound native receptor conformation.

The GluN1-5F11 interface is largely mediated by bulky aromatic residues in 5F11 and the residues in the R2 lobe of GluN1 ATD (Fig. 5C). The hydroxyl groups of Y58 (tyrosine-58) and Y124 in the variable domain of the light chain (VL), together with Y53 and Y126 in the variable domain of the heavy chain (VH), form hydrogen bonds with N385 (asparagine-385), K360 (lysine-360), and L361 (leucine-361) in GluN1 at distances of ~2.7 to 3.0 Å. In addition, Y72 in the V_H_ and W51 (tryptophan-51) and Y126 in the VL domains make van der Waals contacts with L361, N385, and K360 in GluN1. Notably, K360 in GluN1 is extensively involved in these interactions, suggesting a key role in 5F11 recognition (fig. S10, A and B). At the GluN2A-3D2 interface (Fig. 5D), the guanidinium group of R76 (arginine-76) in the VH forms extensive hydrogen bonds with the main chains of E315 (glutamate-315) and A316 (alanine-316) in GluN2A, at distances of ~3.3 Å. R94 in GluN2A also forms a salt bridge with E54 and van der Waals contacts with W52 in the VH of 3D2 (fig. S10, C and D). Interactions between Y51, Y52, and R116 in the VL of 3D2 and D88 (aspartate-88) and L89 in GluN2A further augmented the GluN2A-3D2 binding.

Comparison of the GluN1 ATD structures in the pFab_1_-bound and 5F11-bound states revealed substantial overlap in their binding sites within the GluN1 R2 lobe (Fig. 5E). The GluN1-5F11 interface has a calculated buried surface area of ~739 Å^2^, with residues between β8 and α9, and between β10 and the ATD-LBD linker, primarily involved in the interaction. These structural elements are similar to those involved in the GluN1-pFab_1_ interactions described above (Fig. 2D), suggesting that these regions of the GluN1 ATD are targeted by NMDAR autoantibodies.

### Anti-NMDAR autoantibodies target small regions within the GluN1 ATD

**Fig. 6.**
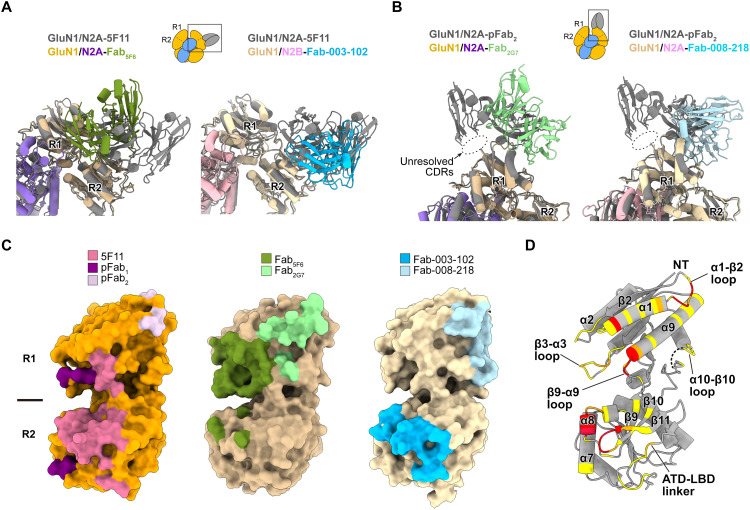
Anti-NMDAR autoantibodies target small regions within the GluN1 ATD. (**A**) Superposition of the native GluN1/GluN2A-5F11 structure with GluN1/GluN2A-Fab_5F6_ and GluN1/GluN2B-Fab-003-102 structures, all of which exhibit “side”’ binding to the receptors. (**B**) Superposition of the GluN1/GluN2A-pFab_2_ structure with GluN1/GluN2A-Fab_2G7_ and GluN1/GluN2A-Fab-008-218 structures, all of which exhibit “top” binding to the receptors. (**C**) Surface mapping of GluN1 ATDs highlighting the antigenic epitopes recognized by each Fab, as determined from the indicated complex structures. (**D**) Residues on the GluN1 ATD that are commonly targeted by autoantibodies demonstrate antigenic hotspots in anti-NMDAR encephalitis. Color scheme: residues targeted by single (yellow), two (orange), or three or four (red) autoantibodies. The unresolved structure is shown as a dotted line.

Recent studies have reported NMDAR structures in complex with patient-derived mAbs, providing additional models of NMDAR-autoantibody interactions (*22*, *23*). These antibodies also target the GluN1 ATD and exhibit two distinct binding modes, binding either to the “side” or on the “top” of the receptor. Structural comparisons of antibody-bound NMDARs reveal that 5F11, Fab_5F6_, and Fab-003-102 recognize the “side” of the GluN1 ATD. Specifically, 5F11 and Fab-003-102 interact with the R2 lobe, whereas Fab_5F6_ primarily binds to the R1 lobe (Fig. 6A). In contrast, pFab_2_, Fab_2G7_, and Fab-008-218 bind to the “top” of the GluN1 ATD through exclusive interactions with the R1 lobe (Fig. 6B). Overall, the side-binding antibodies exhibit larger interface areas (~730 to 941 Å^2^) than the top-binding antibodies (~568 to 631 Å^2^), indicating more extensive contacts with the receptor. Surface mapping of antibody binding footprints on the GluN1 ATD further reveals a high degree of epitope overlap across these antibodies (Fig. 6C and table S1). In the 5F11-bound structure, residues on α1, between α8 and α9, and between β10 and the ATD-LBD linker of GluN1 interact with the antibody, closely resembling those observed in the Fab-003-102 complex. Fab_5F6_ shows extensive contacts with residues on α1, the β3-α3 loop, and α9 of GluN1 R1 lobe. For the top-binding antibodies, interactions consistently involve residues on α1 and the α1-β2 loop of the GluN1 R1 lobe.

Collectively, residues on α1, the α1-β2 loop, α8, and the β10-β11 loop of GluN1 frequently serve as key binding sites for autoantibody recognition (Fig. 6D and fig. S11). Especially, charged residues with bulky side chains such as histidine, lysine, arginine, and glutamate are common targets. These regions display low sequence homology with the corresponding segments in GluN2A and GluN2B subunits (fig. S11), suggesting that GluN1-specific elements function as critical target epitopes in anti-NMDAR autoimmune disorders.

## DISCUSSION

The structures of GluN1/GluN2A receptors in complex with polyclonal Fabs derived from anti-NMDAR encephalitis mice revealed that the IgG repertoire primarily targets the GluN1 ATD. The polyclonal Fabs exhibited two distinct binding modes: pFab_1_ bound laterally to the receptor, parallel to the membrane plane, whereas pFab_2_ bound along the vertical axis. Based on these orientations, we speculate that the IgG corresponding to pFab_1_ recognizes two NMDARs side by side via their bivalent binding sites. In contrast, IgG of pFab_2_ likely engages at binding sites on GluN1 ATDs within a single receptor. Supporting this model, the GluN1-specific 5F11 mAb, which targets the R2 lobe similarly to pFab_1_, bound two GluN1/GluN2A receptors simultaneously, consistent with a mechanism in which autoantibodies mediate receptor cross-linking at synaptic membranes (*8*). Using the monoclonal Fab fragments targeting GluN1 (5F11 Fab) and GluN2A (3D2 Fab) subunits, we purified native NMDARs from mouse brain and resolved the molecular interactions at the autoantibody-receptor interfaces in the most abundant NMDAR subtype, triheteromeric GluN1/GluN2A/GluN2B receptors. By comparing multiple NMDAR-autoantibody complex structures, including the recently reported mAb complexes (*22*, *23*), we defined small, solvent-exposed regions on the GluN1 ATD as antigenic “hotspots” in anti-NMDAR encephalitis.

### The GluN1 ATD in anti-NMDAR encephalitis

The ATDs of NMDARs modulate channel activity through conformational rearrangements of their bilobed structure, composed of R1 and R2 lobes (*1*, *18*). The GluN2 ATDs undergo more dynamic structural changes across different gating states, whereas the GluN1 ATD retains a relatively rigid conformation (*19*, *47*, *48*). Our structural analysis shows that mouse autoantibodies in our active immunization mouse model predominantly target the GluN1 subunit. Of note, the antibody-bound GluN1 ATD conformation remained nearly identical compared to the unbound state [pFab_1_-bound versus Protein Data Bank (PDB) ID 6MMP (*19*): Cα RMSD = 0.029 Å]. This observation provides a structural rationale that autoantibodies from mouse models and patients with anti-NMDAR encephalitis do not acutely block open NMDAR channels (*16*, *49*), but can trigger receptor internalization. This is consistent with our previous electrophysiological recordings, in which rapid perfusion of antibodies onto whole-cell clamped cultured hippocampal neurons had no acute effect on receptor function, whereas a reduction in total current was observed after 24 hours, likely due to receptor internalization (*16*).

Although the IgG repertoire in anti-NMDAR encephalitis mice mostly targeted “rigid” regions of the GluN1 ATD, some antibodies may bind flexible loops or linear epitopes that are not resolved in cryo-EM due to conformational mobility. This limitation may prevent the identification of all potential binding modes associated with anti-NMDAR autoantibodies. Similarly, during the development of mAbs, we excluded antibodies that recognized denatured receptor conformations (*26*), introducing a bias against antibodies targeting linear epitopes on the receptor.

Previously, we found that the −Sign mice nonetheless showed histological evidence of cell infiltration and neuroinflammation (*16*). Consistent with these observations, we detected high-affinity antibody species derived from the −Sign mice. Although our analysis primarily focused on severely affected mice displaying clinical signs and their antibodies, future investigation into the implications of antibodies from −Sign mice will be informative.

Autoantibodies against GluN2A and GluN2B subunits have been reported in patients with SLE and schizophrenia (*10*, *50*, *51*). In lupus models in particular, such antibodies recognize a conserved pentapeptide sequence (DWEYS) in the GluN2A and GluN2B ATDs, and are reported to induce acute changes in NMDAR-mediated currents and excitotoxicity (*51*, *52*). However, the pathogenic implications of GluN2-targeting autoantibodies in anti-NMDAR encephalitis remain uncertain. Our cryo-EM analysis of pAb-bound GluN1/GluN2A receptors did not reveal any discernible GluN2 antibody-bound classes. The solvent-accessible surface area of the GluN1 ATD (20,106 Å^2^) shows only a subtle difference from that of the GluN2A ATD (19,877 Å^2^). However, the GluN2A ATD is positioned closer to the central axis of the receptor (B/D positions), where the R2 lobes adopt a tightly packed conformation. In contrast, the GluN1 ATD resides farther from the central axis (A/C positions), exposing more surface to the environment to accommodate large molecular complexes. Furthermore, GluN1 is ubiquitously expressed in the brain and is essential for receptor assembly, with two GluN1 subunits required per receptor. We speculate that this spatial arrangement, together with the high abundance of GluN1, may partly contribute to the preferential targeting of the GluN1 ATD in anti-NMDAR encephalitis.

### Pathogenic implications of autoantibody binding

It has been widely accepted that clustering and internalization of NMDARs result in receptor hypofunction as a potential contributing factor to disease manifestations. However, NMDAR autoantibodies may also alter NMDAR extrasynaptic trafficking (*53*). Furthermore, a patient-derived antibody that binds to GluN1 ATD reduced NMDAR-mediated synaptic current after 30 min of incubation (*23*), consistent with antibody-mediated receptor internalization. In particular, IgG-008-218 can cross-link two NMDARs through R1 lobe binding. Although we do not yet know which effects of autoantibodies are directly pathogenic, pathogenesis appears to require conformationally restricted epitopes, as immunization with linear peptides has generally not caused signs of encephalitis. In our active immunization mouse model, neuroinflammation was prominent with infiltration of B cells and T cells across the blood-brain barrier and into regions such as the hippocampus (*16*). Similarly, in some patients, CD20^+^ B cells have been found in the CSF and brain (*54*). The effectiveness of rituximab, the anti-CD20 mAb, in clinical cases of anti-NMDAR encephalitis (*55*) indicates the role for antibody-producing B cells in pathogenesis. Intriguingly, it has been reported that some naïve B cells in healthy individuals produce antibodies against GluN1 ATD (*56*), implying incomplete central B cell tolerance or involvement of innate immune responses.

The current treatment options for patients with anti-NMDAR encephalitis are limited to nonspecific immunotherapies (*8*) that can be associated with substantial morbidity. Targeting of the GluN1 ATD may be useful to mask the binding sites of autoantibodies in these patients (*57*). Furthermore, synthetic binders such as small molecules or engineered proteins could be designed to compete with autoantibodies by occupying the same receptor surface. Alternatively, such molecules could bind directly to the autoantibodies, preventing their association with the receptor and thereby mitigating downstream pathogenic effects. In this context, our structural analysis on GluN1-targeting autoantibodies provides potential therapeutic approaches for anti-NMDAR encephalitis.

## MATERIALS AND METHODS

### Expression and purification of GluN1/GluN2A receptors

Expression and purification of *Rattus norvegicus* GluN1/GluN2A receptors followed previously described protocols (*19*, *58*). Briefly, human embryonic kidney (HEK) 293T/17 cells (American Type Culture Collection CRL-11268) were grown in suspension and transduced with P2 BacMam viruses encoding the C-terminal truncated GluN1 (residues 1 to 847) and GluN2A (residues 1 to 866) at a multiplicity of infection of 2:2:1 (GluN1:GluN2A:cells) and incubated at 37°C. Twelve hours posttransduction, 10 mM sodium butyrate was added to the cultures and the temperature was shifted to 30°C. Cells were harvested 60 hours posttransduction, resuspended in tris-buffered saline (TBS; 20 mM tris-HCl and 150 mM NaCl, pH 8.0), and solubilized for 2 hours at 4°C in TBS supplemented with 1% lauryl maltose neopentyl glycol (LMNG), 2 mM cholesteryl hemisuccinate (CHS), 0.8 μM aprotinin, leupeptin (2 μg/ml), 2 mM pepstatin A, and 1 mM phenylmethylsulfonyl fluoride. The solubilized material was applied to Strep-Tactin resin and eluted in TBS containing 0.02% (w/v) LMNG, 0.2 mM CHS, and 5 mM desthiobiotin. For receptors subjected to proteoliposome preparation, the eluate was further treated with thrombin at a 1:50 (w/w) thrombin-to-protein ratio and incubated at room temperature for 2 hours to cleave GFP from the receptor. Either GFP-cleaved or noncleaved receptor was then purified by SEC on a Superose 6 Increase 10/300 GL column in TBS supplemented with 0.02% (w/v) LMNG and 0.2 mM CHS. Peak fractions were pooled and concentrated to 4 to 5 mg/ml using a 100 kDa molecular weight cutoff centrifuge protein concentrator.

### Proteoliposome preparation and immunization

Purified receptors were reconstituted into proteoliposomes for immunization as previously described (*26*). Liposomes were prepared using a lipid mixture consisting of asolectin:cholesterol:brain polar lipid:lipid A in a 24:7:8:1.5 (w/w) ratio. Approximately 40 mg of total lipids were dissolved in 5 ml of chloroform in a glass round-bottom flask. The chloroform was evaporated using a rotary evaporator, and the dried lipid film was rehydrated in 10 ml of TBS (pH 8.0). The lipid suspension was subjected to 10 to 15 freeze-thaw cycles by altering immersion in liquid nitrogen and a 40°C water bath. Liposomes were formed by extruding the resuspended lipids through two 0.2-μm filters 10 times or until the solution appeared translucent. The liposomes were then pelleted by centrifugation at 100,000*g* for 45 min and resuspended in TBS (pH 8.0) at a concentration of 40 mg/ml.

Purified receptor protein was added to the liposomes at a 1:80 (w/w) protein-to-lipid ratio in the presence of 0.5% (w/v) LMNG and incubated on ice for 30 min. Excess detergent was removed by incubation with 200 to 250 mg of Bio-Beads per 1 ml of proteoliposomes containing ~500 μg of protein. Subsequently, proteoliposomes were collected and pelleted by centrifugation at 100,000*g* for 45 min. The proteoliposome pellet was gently resuspended in phosphate-buffered saline (PBS) and used to immunize C65BL6/J female adult mice (JAX no. 000667). Mice received a right inguinal subcutaneous injection of proteoliposomes (25 μg of NMDAR protein per 200 μl) followed by a booster immunization 2 weeks later. Littermate controls were injected with empty liposomes with the same protocol. All proteoliposome and empty-liposome injected mice survived to the 6-week time point. At 6 weeks postimmunization, a blinded observer scored the mice in home cage using a 0 to 5 scoring scale to assess signs of encephalitis: hyperactivity (0 = normal exploratory movement, 1 = continuous running); circling within the long body axis (absent = 0, 1 = present); hunched back and lethargy (0 = absent, 1 = present); clinical seizure (0 = absent, 1 = present); and death (0 = absent, 1 = present) as previously described (*16*). Encephalitis scores were used to group mice based on the presence or absence of encephalitis signs into “+Sign” or “−Sign” for further analysis.

### Purification of autoimmune IgGs and Fab fragments

For IgG purification, serum was collected from the proteoliposome-injected mice following the previously described protocol (*26*). IgG was isolated using Protein A/G beads (Pierce) according to the manufacturer’s protocol. IgG concentration was estimated using a NanoDrop spectrophotometer assuming an extinction coefficient of 13.7 at 280 nm for a 1% (10 mg/ml) IgG solution. The purified IgG was tested for binding to GFP-fused GluN1/GluN2A receptor protein using FSEC (*24*) in TBS (pH 8.0) supplemented with 0.02% (w/v) LMNG.

For Fab fragment generation, purified IgG was digested with papain at a 1:30 (w/w) papain-to-antibody ratio in the presence of 10 mM cysteine and incubation for 2.5 hours at 37°C. The digestion was quenched by adding 30 mM iodoacetamide, followed by incubation at room temperature for 20 min in the dark. Fab fragments were separated from Fc fragments by loading the reaction mixture onto Protein A/G beads, and the flow-through containing Fab fragments was collected. Fab fragments were then concentrated to the desired volume using a 30-kDa molecular weight cutoff centrifuge protein concentrator for SEC.

### Negative-stain EM for GluN1/GluN2A-5F11 mAb complex

Generation and validation of anti-GluN1 5F11 mAb were previously described (*26*). 5F11 mAb was mixed with purified GluN1/GluN2A receptors in a 1:1 molar ratio and subjected to SEC on a Superose 6 Increase 10/300 GL column in TBS supplemented with 0.02% (w/v) LMNG and 0.2 mM CHS. Peak fractions corresponding to the GluN1/GluN2A-5F11 mAb complex were pooled and concentrated to ~0.035 mg/ml. A 400-mesh Cu grid with continuous carbon support (Ted Pella) was glow-discharged for 30 s at 15 mA. A 3.5-μl aliquot of the sample was applied to the carbon side of the grid, followed by three washes with double-distilled water and negative staining with 0.75% uranium formate. Grids were imaged using an FEI Tecnai 12 transmission electron microscope (TEM) equipped with an Eagle 2K TEM charge-coupled device multiscan camera, at a magnification of 49,000× [4.40 Å per pixel (Å/pix)].

### Isolation of native NMDARs from mouse brains

Native NMDARs were isolated from C57BL/6 male and female mice (Charles River) aged 28 to 42 days. Fifty brains were homogenized in ice-cold TBS (pH 8.0) with protease inhibitors and disrupted by sonication (90 s total; 3 s on, 9 s off) on ice. The homogenates were solubilized with 2% (w/v) digitonin for 2 hours at 4°C, followed by centrifugation at 200,000*g* for 1 hour. The resulting supernatant was incubated with excess anti-GluN1 5F11 Fab (*26*) fused to a GFP-3xFLAG and anti-FLAG M2 resin for 1 hour at 4°C with nutation. The mixture was loaded onto a gravity column and washed with TBS (pH 8.0) containing 0.1% (w/v) digitonin. Bound receptors were eluted by incubation with the same buffer supplemented with 3xFLAG peptide (0.1 mg/ml) for 15 min on ice. The eluate was then concentrated and incubated with excess anti-GluN2A 3D2 Fab for 30 min on ice, followed by SEC using a Superose 6 Increase 10/300 GL column in TBS (pH 8.0) with 0.1% (w/v) digitonin. Peak fractions were pooled and concentrated using a 100-kDa molecular weight cutoff centrifuge protein concentrator.

### Western blot analysis

Protein samples were mixed with loading buffer supplemented with 0.1 M dithiothreitol and incubated at 50°C for 10 min. Samples were then separated by SDS-PAGE using 4 to 20% gradient gels and transferred to polyvinylidene fluoride membranes. Immunoblotting was performed using the following primary antibodies: anti-GluN1 (1:600; Alomone Labs, AGC-001), anti-GluN2A (1:600; Alomone Labs, AGC-002), anti-GluN2B (1:600; Alomone Labs, AGC-003), anti-GluN2C (1:200; Alomone Labs, AGC-018), anti-GluN2D (1:200; Alomone Labs, AGC-020), and anti-GluN3A (1:200; Alomone Labs, AGC-030). Membranes were then incubated with IRDye 800CW goat anti-rabbit (LI-COR 926-32211) or goat anti-mouse (LI-COR 926-32210) IgG secondary antibody at a dilution of 1:20,000. Protein bands were visualized using a LI-COR Odyssey CLx imaging system.

### Grid preparation, cryo-EM, and single-particle analysis

Purified GluN1/GluN2A receptors were incubated with polyclonal Fabs at an estimated 1:140 molar ratio or with the 3D2 Fab at a 1:3 molar ratio on ice for 1 hour, followed by SEC purification. The native NMDAR-5F11 complex were incubated with excess 3D2 Fab on ice for 1 hour and subjected to SEC purification. Peak fractions were collected, analyzed by SDS-PAGE, and concentrated in the presence of 1 mM EDTA.

For grid preparation, Quantifoil R2/1 200 mesh Au holey grids were glow-discharged for 1 min at 15 mA and applied with the GluN1/GluN2A-3D2 Fab complex at 4 to 5 mg/ml in solution that was supplemented with 1 mM glycine, 1 mM glutamate, and 0.1 mM fluorinated octyl-maltoside (FOM). Quantifoil R2/1 200 mesh Au grids with a 2 nm carbon layer were glow-discharged for 30 s at 15 mA and applied with either the GluN1/GluN2A-pFab or the native NMDAR-5F11/3D2 complex at 0.5 to 0.75 mg/ml, supplemented with 1 mM glycine, 1 mM glutamate, and 0.1 mM FOM. For vitrification, 3 μl of protein solution was applied to the grid, blotted for 3 s with a blot force of 0, and then plunge-frozen in liquid ethane using an FEI Vitrobot Mark IV at 16°C and 100% humidity. A blotting wait time of 0 s was used for holey grids and 25 s for 2-nm carbon-layered grids.

The GluN1/GluN2A-pFabs dataset was collected on a 300-keV FEI Titan Krios equipped with a Gatan K3 camera and BioQuantum energy filter in super-resolution mode, at a magnification of 29,000× (0.798 Å/pix). Images were acquired using SerialEM with a defocus range of −1.4 to −2.5 μm, a dose rate of ~15 e^−^ pix^−1^ s^−1^, and a total dose of ~45 e^−^/Å^2^ over 40 frames. The native NMDAR-5F11/3D2 dataset was collected on a 300-keV FEI Titan Krios with a Gatan K3 camera, BioQuantum energy filter in super-resolution mode at a magnification of 64,000× (1.394 Å/pix), with a defocus range of −1.0 to −3.0 μm, a dose rate of ~8 e^−^ pix^−1^ s^−1^, and a total dose of ~65 e^−^/Å^2^ over 120 frames. The GluN1/GluN2A-3D2 dataset was collected on a 300-keV FEI Titan Krios with a Gatan K3 camera, BioQuantum energy filter in super-resolution mode at a magnification of 105,000× (0.830 Å/pix), with a defocus range of −1.0 to −2.5 μm, a dose rate of ~23 e^−^ pix^−1^ s^−1^, and a total dose of ~50 e^−^/Å^2^ over 45 frames.

Movie alignment, contrast transfer function estimation, particle picking, 2D/3D classification, ab initio 3D reconstruction, nonuniform refinement, and local refinement were performed using CryoSPARC v4 (*59*). Masks for local refinement were generated in UCSF Chimera (*60*). Map resolutions were determined using the gold-standard Fourier shell correlation (FSC) at 0.143 between two half maps in CryoSPARC. Data collection and model refinement statistics are summarized in Table 1. Cryo-EM data processing workflows for the GluN1/GluN2A-pFab, native NMDAR-5F11/3D2, and GluN1/GluN2A-3D2 complexes are shown in figs. S2, S4, and S8, respectively. Figures were prepared with UCSF ChimeraX (*61*).

Structural models of recombinant and native GluN1/GluN2A and GluN1/GluN2B were built based on PDB ID 6MMP (*19*) and 7SAA (*30*), respectively. The triheteromeric GluN1/GluN2A/GluN2B model was built based on this GluN1/GluN2A model with one GluN2A subunit replaced by GluN2B. Initial models of 5F11 and 3D2 Fabs were predicted from their sequences using AlphaFold3 (*62*). The pFab_1_ and pFab_2_ models were generated by adapting the 5F11 structure, replacing the amino acid sequence with unknown residues (UNK) and modeling only the Cα atoms. Model building was performed in Coot (*63*), and real-space refinement was conducted using Phenix (*64*) against the corresponding cryo-EM maps. Contact areas of antibody-receptor interfaces were calculated using PISA (*65*).

### Octet biolayer interferometry analysis

For biolayer interferometry analysis, the Octet Red384 System (ForteBio) was used according to the manufacturer’s protocol. The purified GluN1/GluN2A receptor was diluted to a concentration of 25 μg/ml in running buffer—TBS (pH 8.0) supplemented with 0.02% (w/v) LMNG—and incubated with prehydrated Octet NTA Biosensors (Sartorius, 18-5101) for 600 s. The receptor-coated biosensors were equilibrated for 300 s in running buffer. They were then transferred to wells containing either pFabs or 5F11 Fab-GFP at varying concentrations of 8 to 500 nM or 3 to 200 nM, respectively, with one well containing only running buffer to serve as a reference. Association and dissociation kinetics were measured for 900 and 1800 s, respectively. The data were processed by subtracting the reference signal, aligning the *y* axis to the baseline, and fitting to a 1:1 binding model using “full” global fitting in ForteBio Octet Data Analysis HT software.

### Animal use statement

For native receptor purification, 50 postnatal day 28 to 42 C57BL/6 mice (both male and female) from Charles River Laboratories were used. Native receptor quantity per mouse was estimated via fluorescence from recombinant antibody fragments to determine the minimum required sample size. Mice were housed at 20° to 23°C, 40 to 60% humidity, with a 12:12-hour dark/light cycle. No randomization, blinding, or experimental manipulations were performed. All mice were euthanized under the Oregon Health & Science University Institutional Animal Care and Use Committee (IACUC) protocols, consistent with the recommendations of the Panel on Euthanasia of the American Veterinary Medical Association and carried out only by members of the E.G. and G.L.W. laboratories approved on IACUC protocols TR03_IP00000905 and TR03 IP00000148, respectively.

### Cell line statement

Sf9 cells for baculovirus production and recombinant antibody expression were obtained from Thermo Fisher Scientific (12659017, lot 421973). HEK293T/17 cells for protein expression were from the previous study (*19*). HEK293T/17 cells tested negative for mycoplasma contamination using the CELLshipper Mycoplasma Detection Kit M-100 (Bionique).
