# Supplementary material for: Cryo-EM of autoantibody-bound NMDA receptors reveals antigenic hotspots in an active immunization model of anti-NMDAR encephalitis
Source: Sci Adv. 2026 Jan 14;12(3):eaeb4249. doi: 10.1126/sciadv.aeb4249 (PMC12802853; doi:10.1126/sciadv.aeb4249)
Supplement: Supplementary file 1 — Figs. S1 to S11 Table S1 [file sciadv.aeb4249_sm.pdf]

Science Advances
AAAS

Supplementary Materials for

Cryo-EM of autoantibody-bound NMDA receptors reveals antigenic hotspots in an active immunization model of anti-NMDAR encephalitis

Junhoe Kim *et al.*

Corresponding author: Eric Gouaux, gouauxe@ohsu.edu

Sci. Adv. **12**, eaeb4249 (2026)
DOI: 10.1126/sciadv.aeb4249

This PDF file includes:

Figs. S1 to S11
Table S1

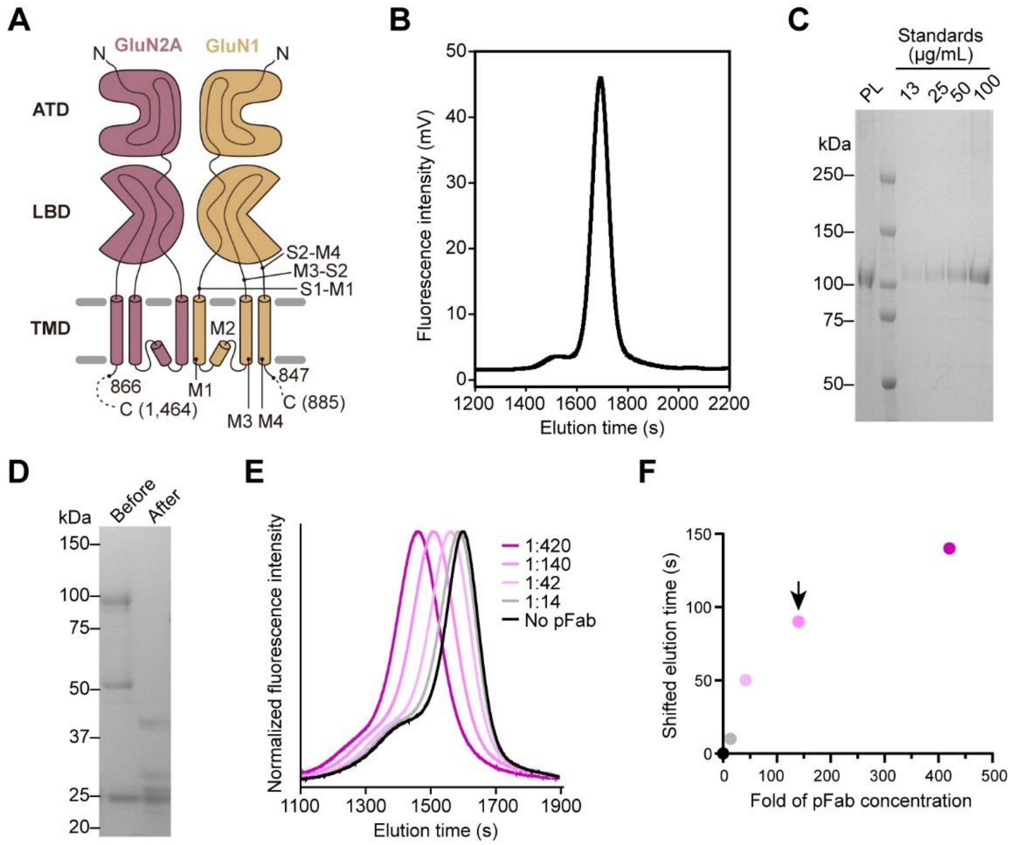

Fig. S1. Production and validation of anti-NMDAR targeting polyclonal antibodies and Fab fragments. (**A**) Schematic representation of *Rattus norvegicus* GluN1/GluN2A expression constructs used in this study. (**B**) Representative FSEC profile of purified GluN1/GluN2A receptors. (**C**) SDS-PAGE gel image showing GluN1/GluN2A receptors reconstituted in proteoliposomes (PL) alongside quantification standards. (**D**) SDS-PAGE gel image of pAbs before and after papain digestion to generate polyclonal Fab fragments (pFabs). (**E**) FSEC profiles of GluN1/GluN2A receptors in complex with the indicated molar excesses of pFabs, showing shifts in the receptor peak position upon pFab binding. (**F**) Plot summarizing the extent of receptor peak shift in **F**. A sub-saturating 1:140 molar ratio (arrow) was selected for subsequent purification of the receptor–antibody complex (see Main text).

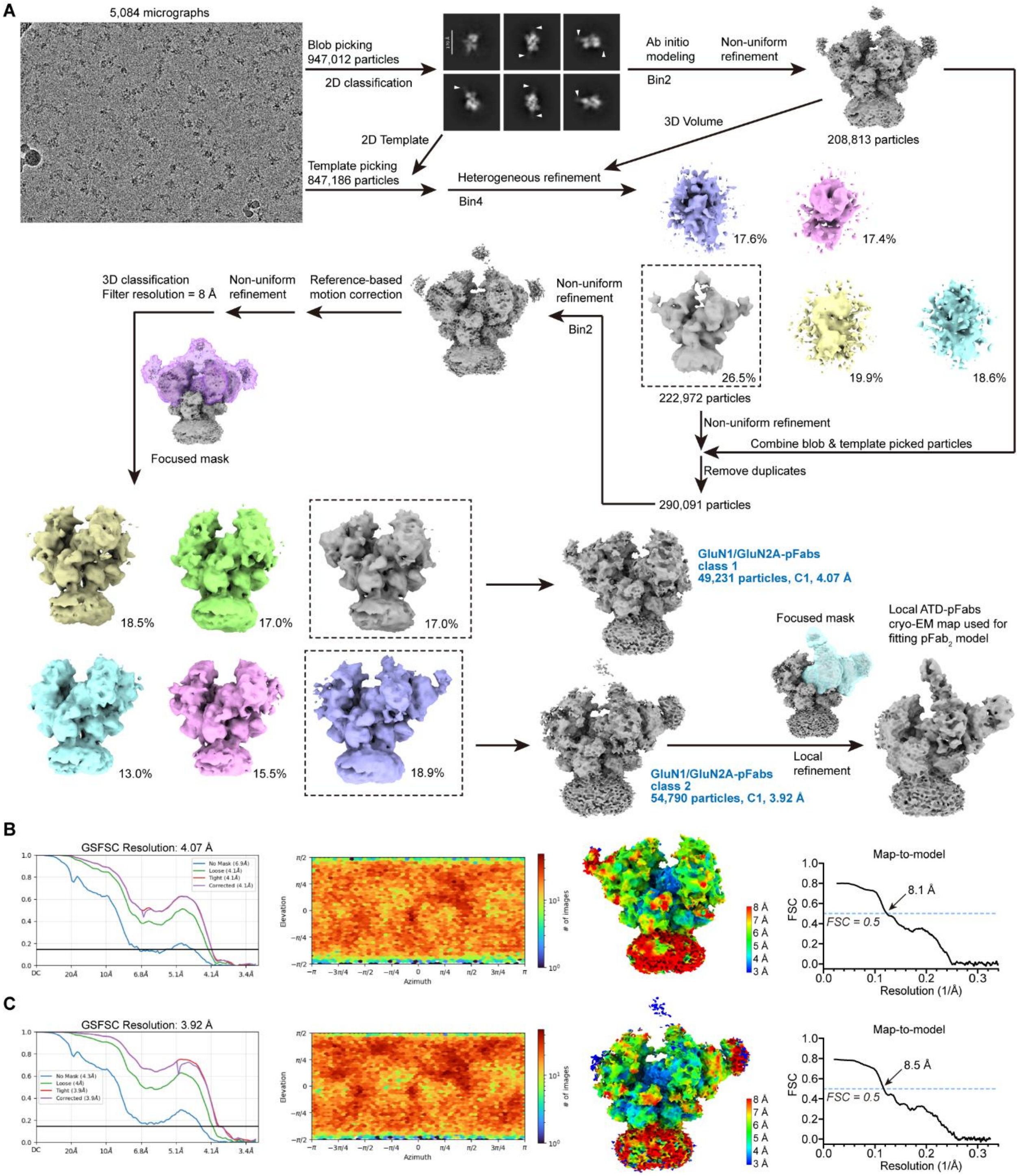

Fig. S2. Single-particle cryo-EM analysis of GluN1/GluN2A-pFab complexes. (**A**) Representative micrograph, 2D and 3D classification images, and image processing workflow. (**B** and **C**) From left to right: gold standard Fourier shell correlation (GSFSC) curves, orientation distribution plot, local resolution estimation, and FSC curve for map-to-model validation of GluN1/GluN2A-pFabs class 1 (**B**) and class 2 (**C**).

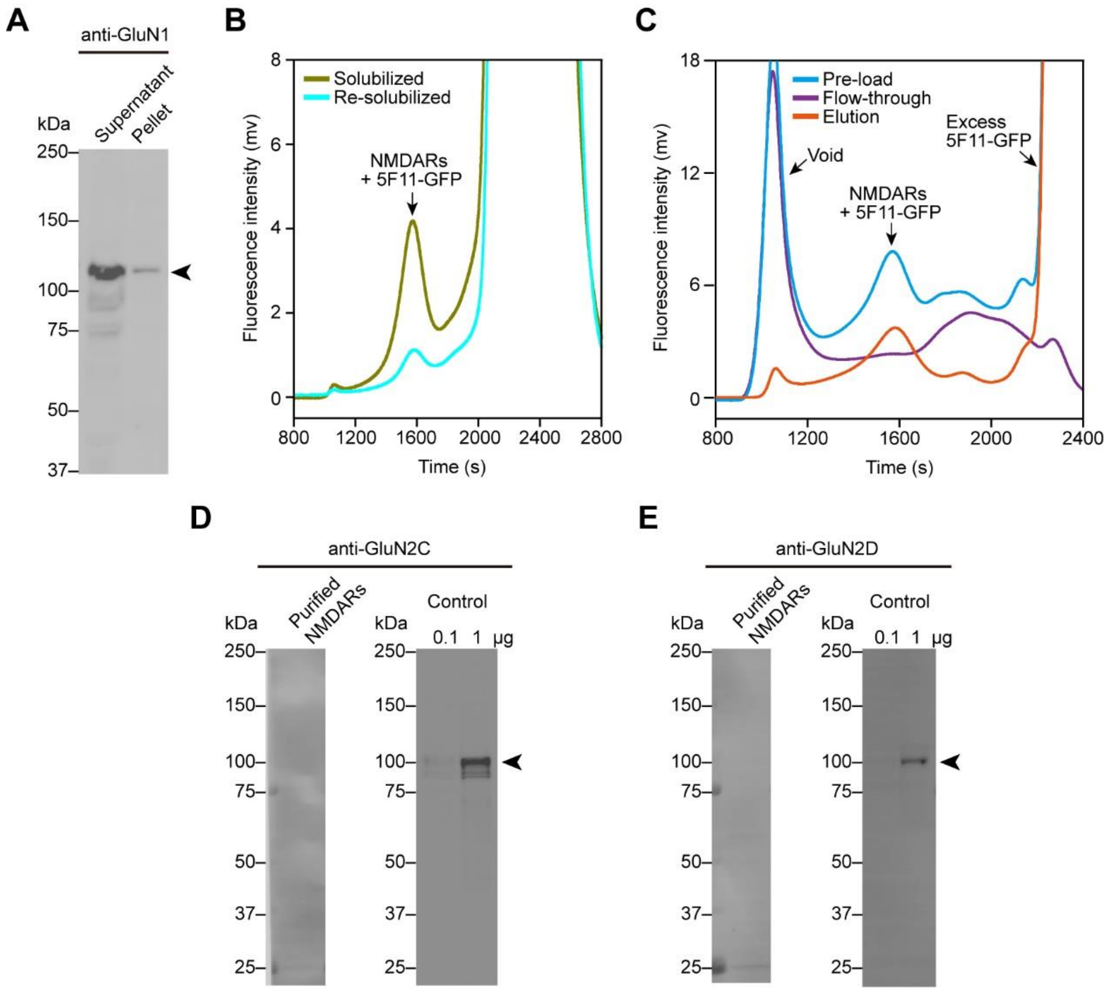

Fig. S3. Biochemical assessment of native NMDAR purification. (**A**) Western blot analysis of GluN1 subunits in the supernatant and pellet fractions after solubilizing mouse brain lysate (see Methods). (**B**) FSEC profiles showing native NMDARs in the samples initially solubilized with digitonin and subsequently re-solubilized from the pellet in **A** using a stronger detergent mixture (0.1% sodium deoxycholate and 0.1% sodium dodecyl sulfate), probed with GluN1-specific antibody 5F11 Fab-GFP. (**C**) FSEC profiles of the pre-load, flow-through, and elution fractions from FLAG affinity purification of native NMDAR–5F11 Fab-GFP complexes. (**D** and **E**) Western blot analyses of GluN2C (**D**) and GluN2D (**E**) subunits from the purified native NMDAR samples, shown alongside quantification standards prepared from recombinant proteins. Arrows indicate the corresponding band of the subunit.

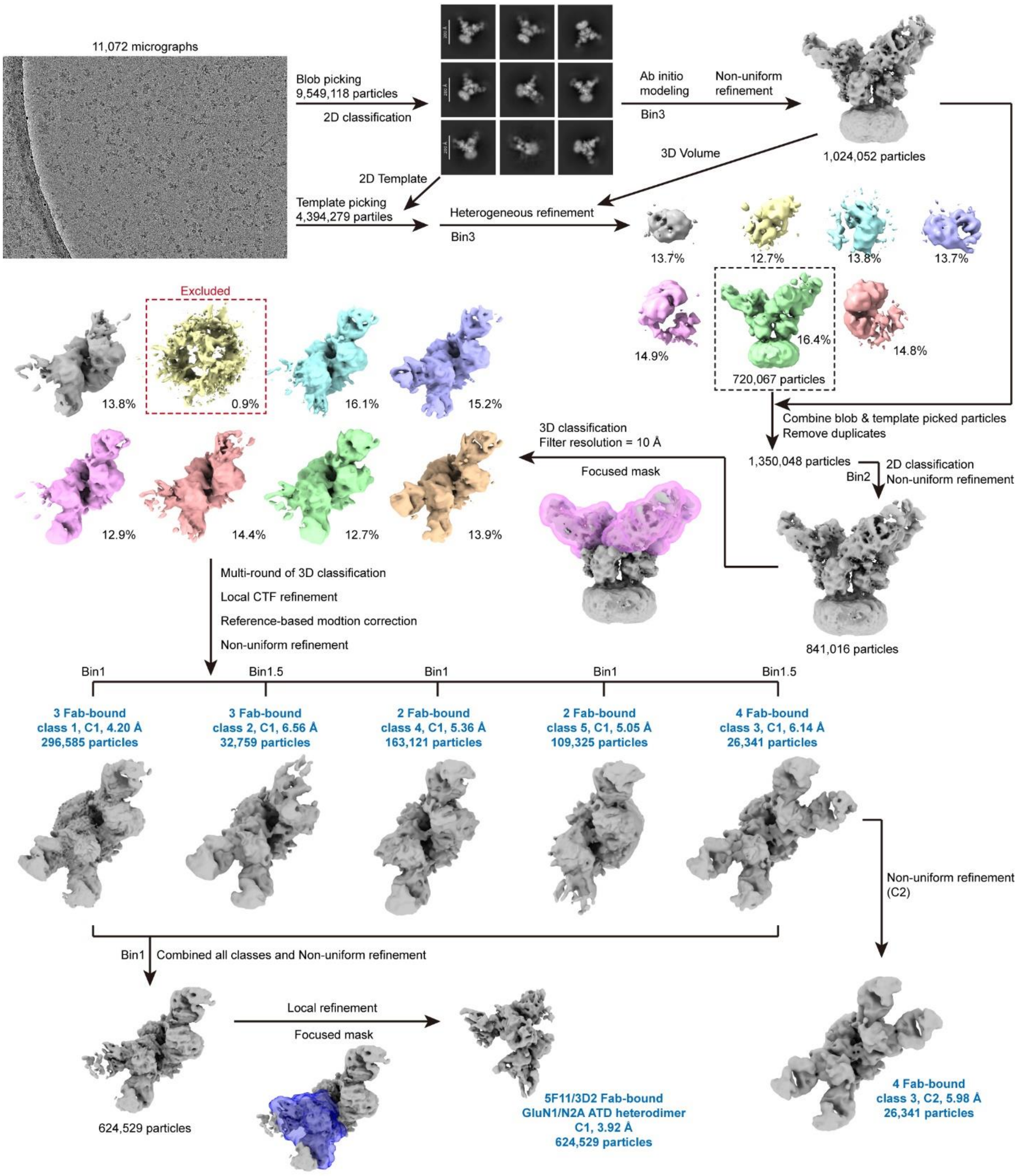

Fig. S4. Single-particle cryo-EM analysis of native NMDARs in complex with 5F11 and 3D2 Fabs. Representative micrograph, 2D and 3D classification images, and image processing workflow are shown.

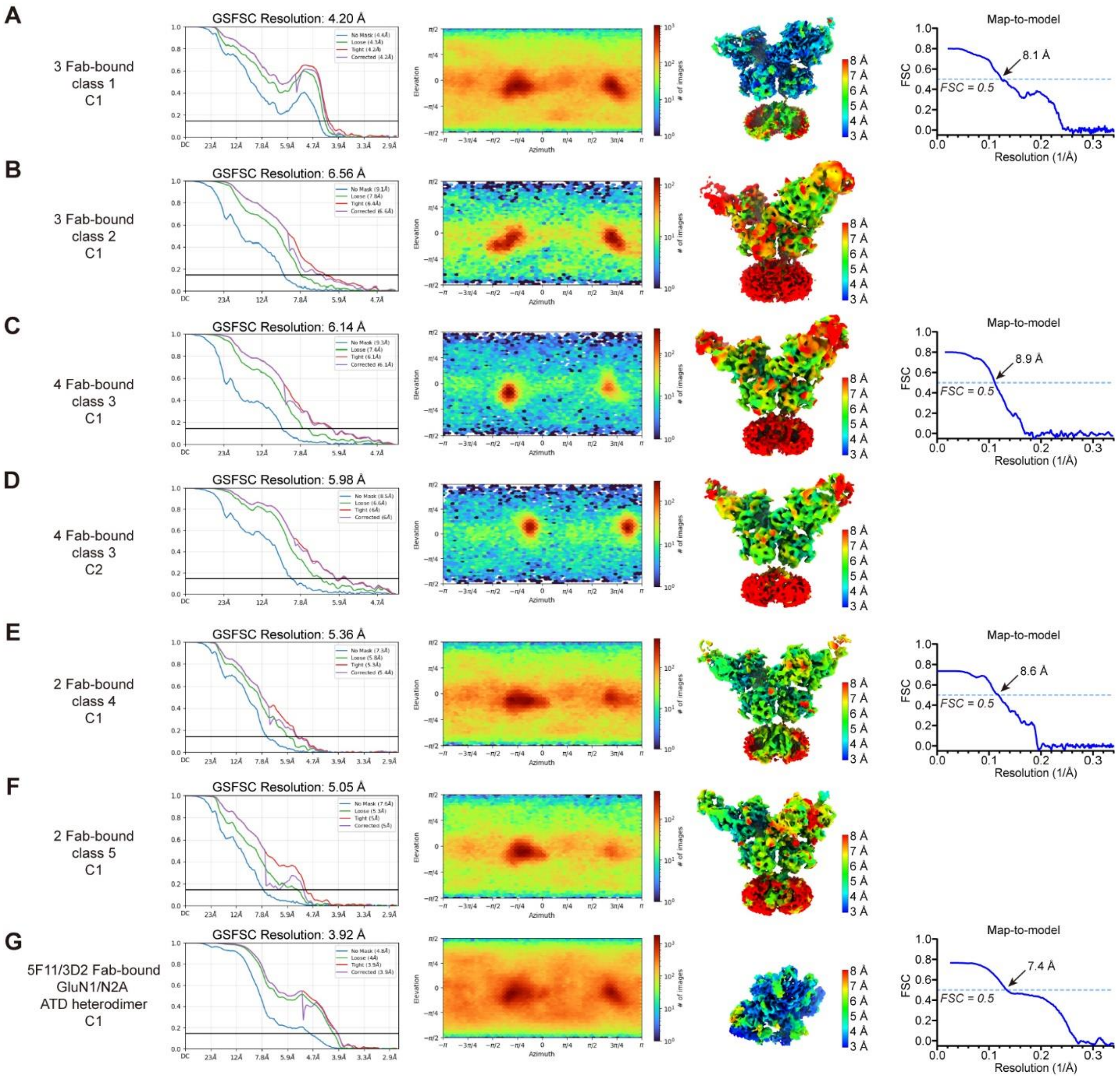

Fig. S5. Validation of cryo-EM maps and models of native NMDAR-Fab complexes. (**A-G**) From left to right: class annotation, gold standard Fourier shell correlation (GSFSC) curves, orientation distribution plot, local resolution estimation, and FSC curve for map-to-model validation of 3 Fab-bound class 1 (**A**), 3 Fab-bound class 2 (**B**), 4 Fab-bound class 3 in C1 symmetry (**C**), 4 Fab-bound class 3 in C2 symmetry (**D**), 2 Fab-bound class 4 (**E**), 2 Fab-bound class 5 (**F**), and the local ATD heterodimer of GluN1/GluN2A-5F11/3D2 (**G**).

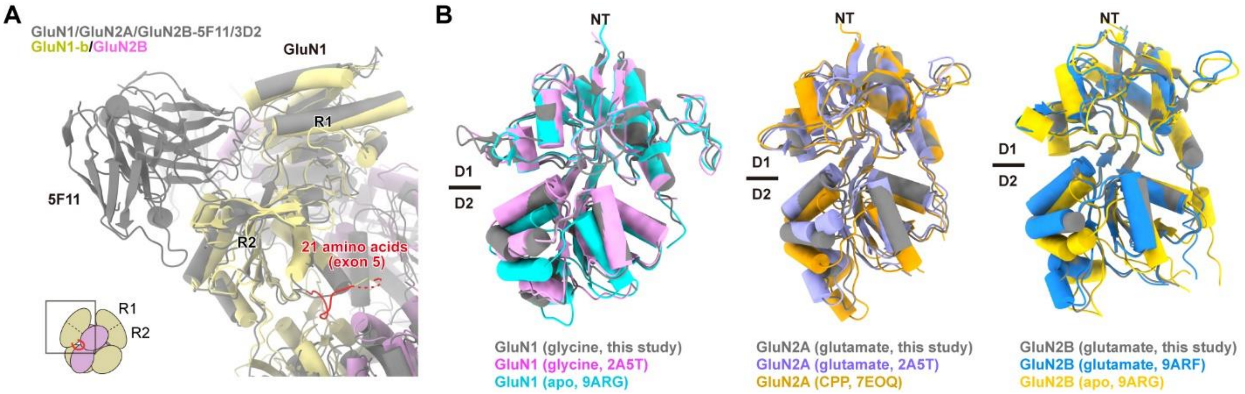

Fig. S6. Structural analysis of 5F11/3D2-bound native GluN1/GluN2A/GluN2B receptor. (**A**) Superposition of the 5F11-bound native GluN1/GluN2A/GluN2B structure with the GluN1-b/GluN2B structure (PDB ID 6CNA), aligned at GluN1 ATDs. The GluN1-b region where 21 amino acids are inserted via alternative splicing of exon 5 was colored red. A dotted line represents missing parts of the structure. (**B**) Comparisons of LBD conformations between this study and previous reports show that the LBDs of native GluN1/GluN2A/GluN2B receptors adopt a glycine- or glutamate-bound closed conformation.

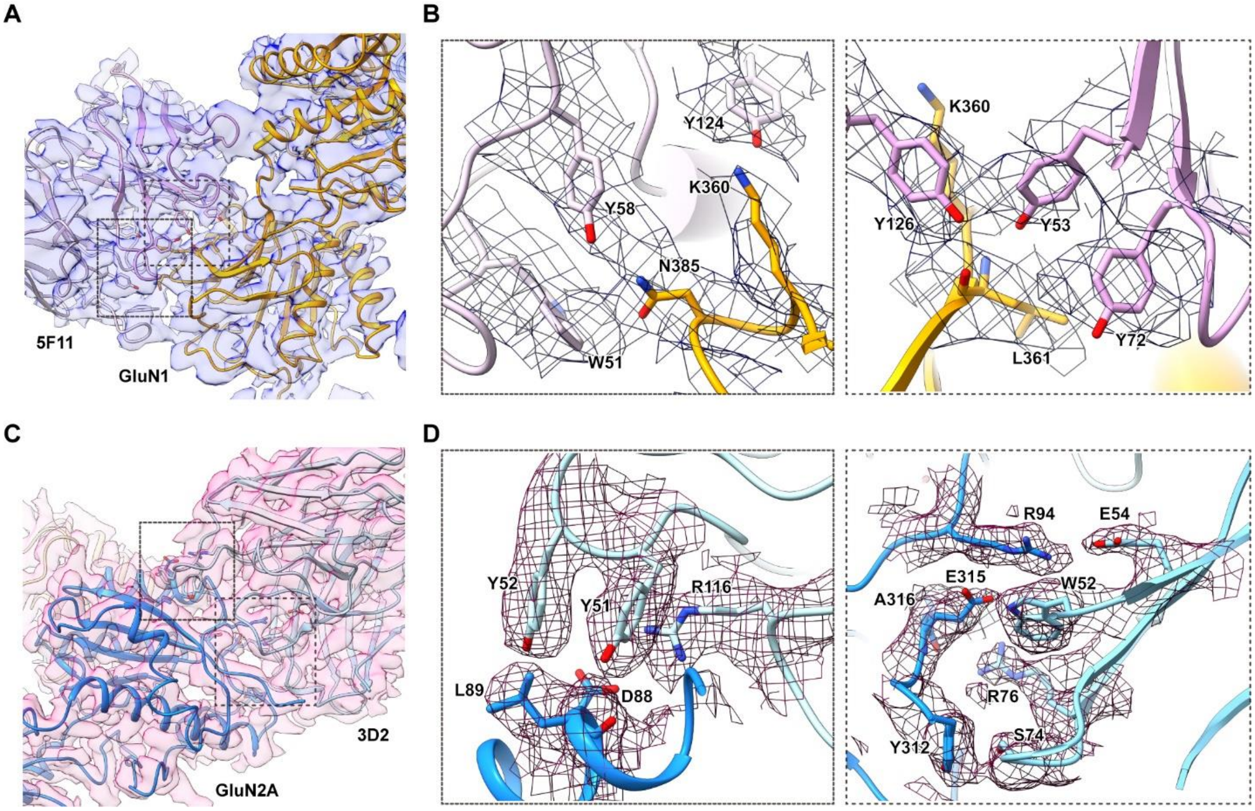

Fig. S7. Cryo-EM densities at the receptor-autoantibody interfaces. (**A**) Structure of the native GluN1-5F11 complex overlaid with its cryo-EM density map (transparent). (**B**) Enlarged views of the boxed areas in **A**, showing residues involved in the GluN1-5F11 interactions. The local filtered map (mesh) is contoured at 0.065 (1.60 σ) within 3 Å of the displayed residues. (**C**) Structure of the recombinant GluN2A-3D2 complex overlaid with its cryo-EM density map (transparent). (**D**) Enlarged views of the boxed areas in **C**, showing residues involved in the GluN2A and 3D2 interactions. The local filtered map (mesh) is contoured at 0.049 (0.60 σ) within 3 Å of the displayed residues.

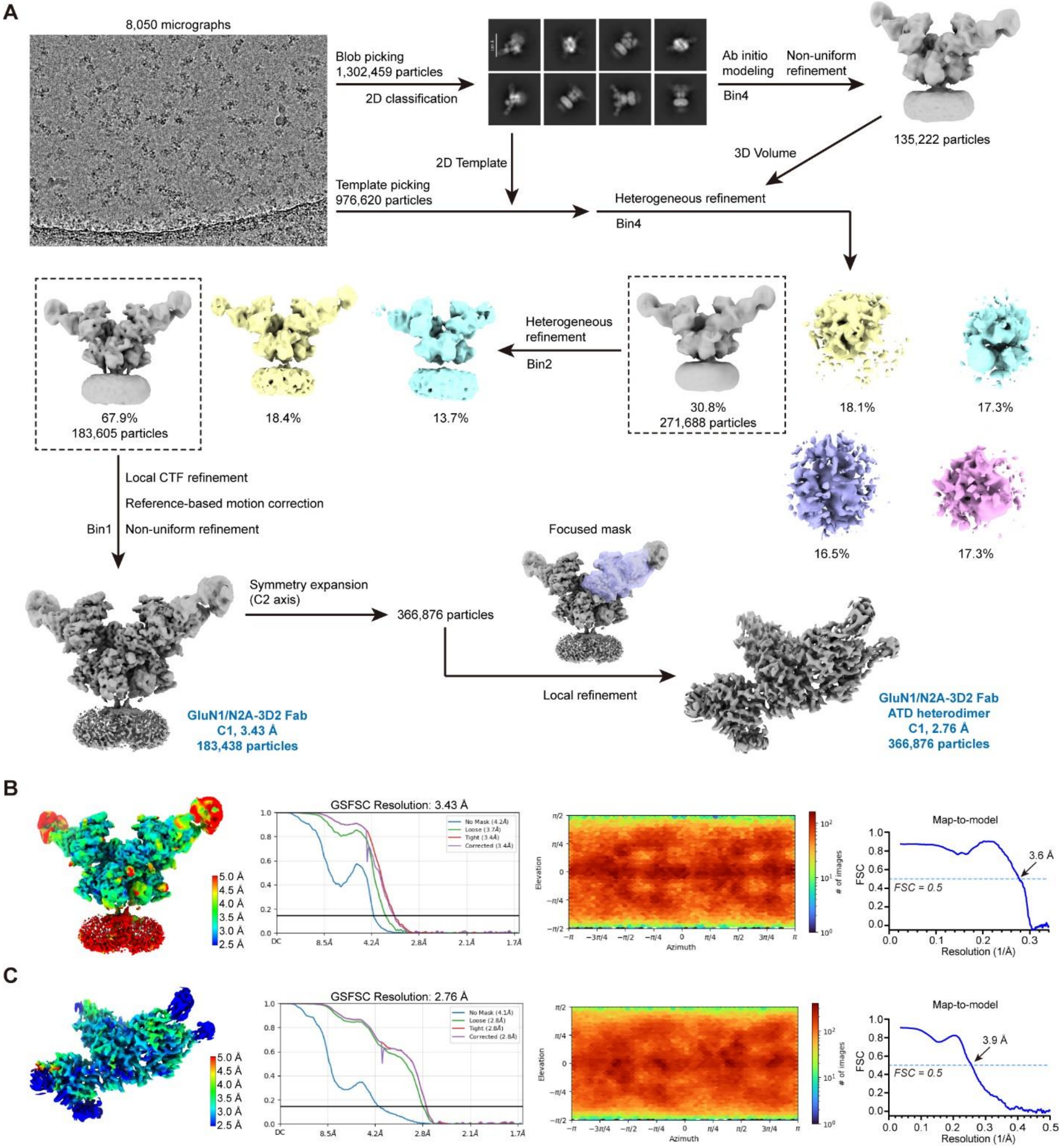

Fig. S8. Single-particle cryo-EM analysis of GluN1/GluN2A-3D2 Fab complex. (**A**) Representative micrograph, 2D and 3D classification images, and image processing workflow. (**B** and **C**) From left to right: local resolution estimation, gold standard Fourier shell correlation (GSFSC) curves, orientation distribution plot, and FSC curve for map-to-model validation for the overall (**B**) and the local ATD heterodimer (**C**) of GluN1/GluN2A-3D2 Fab complex.

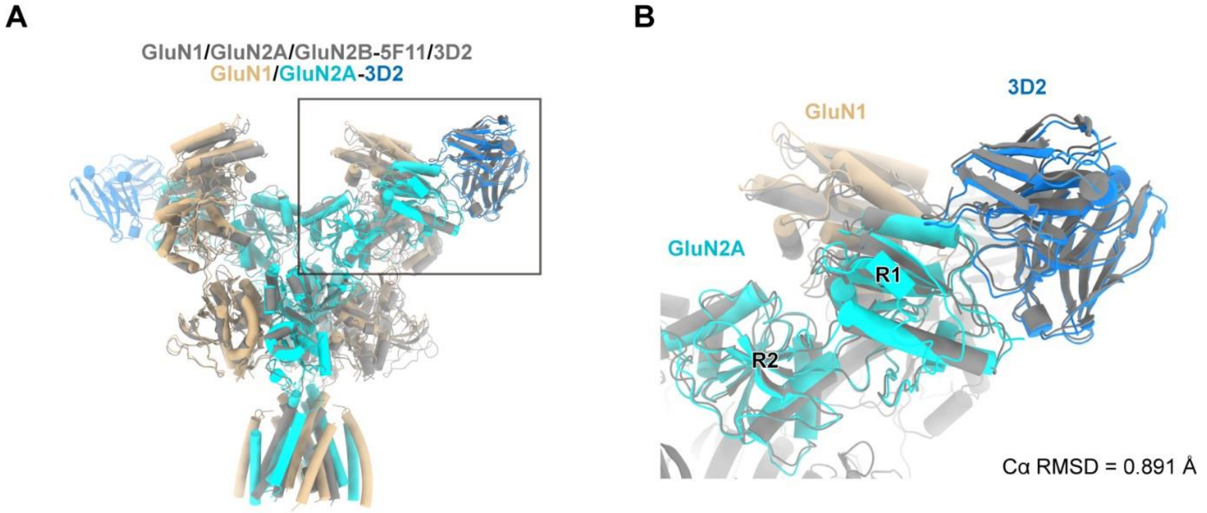

Fig. S9. Comparison of the 3D2-bound GluN2A ATD structures. (**A**) Superposition of the 3D2-bound native GluN1/GluN2A/GluN2B and recombinant GluN1/GluN2A structures, aligned at GluN2A ATDs. The 5F11 Fabs in the native NMDAR complex were omitted for clarity. (**B**) Enlarged view of the boxed region in **A** shows that the conformation of 3D2-bound GluN2A ATDs are largely same between two structures (Cα root-mean-square deviation = 0.891 Å).

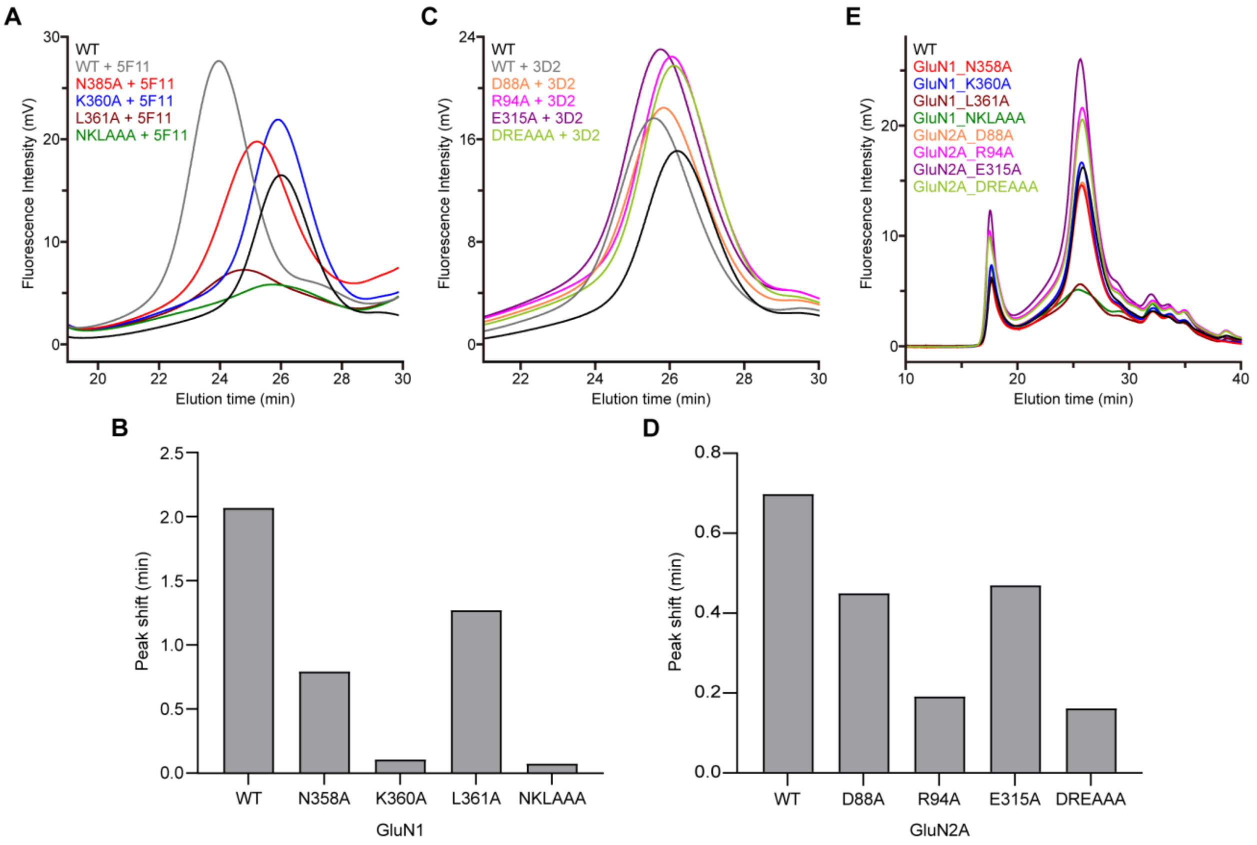

Fig. S10. FSEC shift analysis of GluN1 and GluN2A mutants with Fab binding. (**A**) FSEC profiles of WT and mutant GluN1 expressed with WT GluN2A, in the presence of 100 nM 5F11 Fab-GFP or alone. (**B**) Bar graphs show the FSEC peak shifts of WT and mutant GluN1 upon 5F11 Fab-GFP binding. (**C**) FSEC profiles of WT and mutant GluN2A expressed with WT GluN1, in the presence of 100 nM 3D2 Fab or alone. (**D**) Bar graphs show the FSEC peak shifts of WT and mutant GluN2A upon 3D2 Fab binding. (**E**) FSEC profiles of WT and mutant GluN1 and GluN2A expressed with their WT counterparts in the absence of Fab.

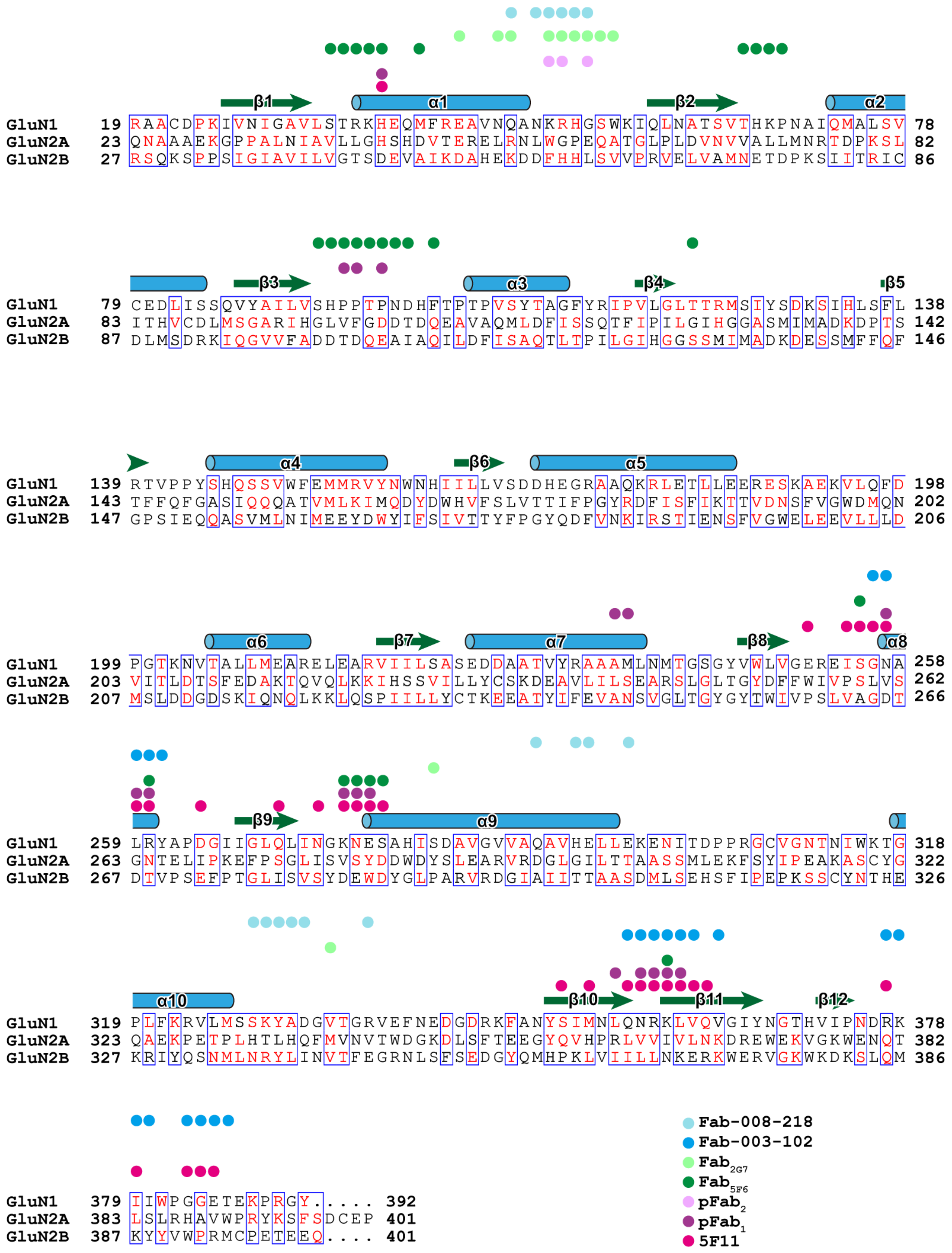

Fig. S11. Sequence alignment of GluN1, GluN2A, and GluN2B ATDs. GluN1 residues involved in the interactions with the indicated autoantibodies are marked, highlighting the locations of antigenic epitopes on the GluN1 ATD. Secondary structural elements are assigned based on the GluN1 sequence.

Table S1. Structural elements and residues involved in GluN1-autoantibody interactions.

Antibody	Interface area (Å^2)	Structural elements (interacting residues)
5F11	738.9	α1 (His38); β8-α8 loop (Glu251, Ile254, Ser255, Gly256); α8 (Asn257, Leu259, Arg260); α8-β9 loop (Asp264); β9 (Gln270); β9-α9 loop (Asn273, Lys275, Asn276); α9 (Glu277, Ser278); β10 (Ser352, Met354, Gln357); β10-β11 loop (Asn358, Arg359); β11 (Lys360, Leu361, Val362, Gln363); ATD–LBD linker (Arg377, Ile379, Gly383, Gly384, Glu385)
pFab$_1$*	333.7	α1 (His38); β3-α3 loop (Pro95, Pro96, Pro98); α7 (Ala236, Met237); α8 (Asn257, Leu259, Arg260); β9-α9 loop (Lys275, Asn276); α9 (Glu277); β10 (Leu356); β10-β11 loop (Asn358, Arg359); β11 (Lys360, Leu361)
pFab$_2$*	25.3	α1-β2 loop (Lys51, Arg52, Gly54)
Fab$_{5F6}$	941.2	β1-α1 loop (Ser34, Thr35); α1 (Arg36, Lys37, His38, Met41); β2-α2 loop (Thr66, His67, Lys68, Pro69); β3-α3 loop (Ser93, His94, Pro95, Pro96, Thr97, Pro98, Asn99, Asp100, Phe102); β4- β5 loop (Thr122); β8-α8 loop (Ser255); α8 (Arg260); β9-α9 loop (Lys275, Asn276); α9 (Glu277, Ser278); β11 (Lys360)
Fab$_{2G7}$	630.5	α1 (Glu44, Asn47, Gln48); α1-β2 loop (Lys51, Arg52, His53, Gly54, Ser55, Trp56); α9 (Ser282); α10-β10 loop (Val334)
Fab-003-102	730.3	β8-α8 loop (Gly256); α8 (Asn257, Leu259, Arg260); α8-β9 loop (Tyr261); β10-β11 loop (Gln357, Arg358, Arg359); β11 (Lys360, Leu361, Val362, Val364); ATD–LBD linker (Arg377, Lys378, Ile379, Ile380, Gly383, Gly384, Glu385, Thr386)
Fab-008-218	568.1	α1 (Gln48); α1-β1 loop (Asn50, Lys51, Arg52, His53, Gly54); α9 (Gln290, His293, Glu294); α9-α10 loop (Glu297); α10-β10 loop (Ser328, Lys329, Tyr330, Ala331, Asp332, Arg337)

*The structures of CDR loops of pFab$_1$ and pFab$_2$ were not modeled; therefore, the calculated interface areas are underestimated, and the listed residues may not represent all possible interactions.
